# Epigenetic-mediated positive feedback loop facilitates the progression of lung adenocarcinoma

**DOI:** 10.1016/j.isci.2025.113339

**Published:** 2025-08-26

**Authors:** Yihang Cheng, Yifeng Luo, Tingting Hu, Qiaoling Ren, Li Xu, Tuo Yi, Yuan Tan, Wei Li, Xuxin Wang, Yaoxiang Sun, Mingzhi Chen, Zhonghua Shen, Bin Zhang, Youhuang Bai, Yue Tao, Zhihong Cao, Deqiang Sun

**Affiliations:** 1 Department of Cardiology of The Second Affiliated Hospital, School of Medicine, Zhejiang University, Hangzhou 310009, China; 2 State Key Laboratory of Transvascular Implantation Devices, Zhejiang University, Hangzhou 310009, China; 3 Heart Regeneration and repair Key Laboratiry of Zhejiang Province, Hangzhou 310009, China; 4 Department of Radiology, the Affiliated Yixing Hospital of Jiangsu University, Yixing 214200, China; 5 Department of research and development, Zhejiang Gaomei Genomics, Hangzhou 310009, China; 6 Information and Communication Engineering, Ningbo University, Ningbo 315800, China; 7 College of Food Science and Engineering, Ningbo University, Ningbo 315800, China; 8 School of Traditional Chinese Medicine, Shanghai University of Traditional Chinese Medicine, Shanghai 201203, China; 9 Department of Cardiovascular Surgery of The Second Affiliated Hospital, School of Medicine, Zhejiang University, Hangzhou 310009, China; 10 Key Laboratory of Biological Targeting Diagnosis, Therapy and Rehabilitation of Guangdong Higher Education Institutes, The Fifth Affiliated Hospital of Guangzhou Medical University, Guangzhou 510300, China; 11 Department of Pathology, the Affiliated Yixing Hospital of Jiangsu University, Yixing 214200, China; 12 Department of Clinical Laboratory, the Affiliated Yixing Hospital of Jiangsu University, Yixing 214200, China; 13 Department of Thoracic and Cardiovascular Surgery, the Affiliated Yixing Hospital of Jiangsu University, Yixing 214200, China

**Keywords:** Biological sciences, Molecular biology, Molecular mechanism of gene regulation

## Abstract

Lung adenocarcinoma (LUAD) remains a leading cause of cancer-related mortality. We performed multi-omics approaches on LUAD samples spanning different stages, establishing a detailed epigenetic landscape. We identified 1,416 regions characterized by hypo-DNA methylation and hyperchromatin accessibility associated with high mortality and recurrence rates, serving as a biomarker panel for LUAD staging and diagnosis (AUC of 0.89 and 0.87 for two-class and multi-class models). Epigenetics-related transcription factors, especially the AP-1 family, were observed at these loci, and transcriptomic profiling indicated persistent activation of the mitogen-activated protein kinase (MAPK) signaling pathway. Hypomethylation and hyperaccessibility of AP-1 binding sites enhance EGFR and FGFBP1 expression, activating the MAPK pathway. This reveals an epigenetic-mediated positive feedback loop, MAPK→AP-1/Epigenetic Modifications→EGFR/FGFBP1→MAPK, in LUAD, highlighting the complex interplay between epigenetic modifications and LUAD progression, offering potential detection and treatment targets. These findings define a high-resolution, multi-stage epigenetic landscape of LUAD, providing a resource for biomarker discovery and mechanistic insight.

## Highlights

• Multi-stage epigenetic map links DNA methylation and accessibility in LUAD
• Epi-TFs mediate crosstalk between epigenetic states and LUAD progression
• 1,416 epigenetic regions predict LUAD stage with high accuracy (AUC 0.89/0.87)
• MAPK–AP-1–EGFR/FGFBP1 loop drives LUAD via epigenetic feedback signaling

## Graphical abstract

**Figure:**
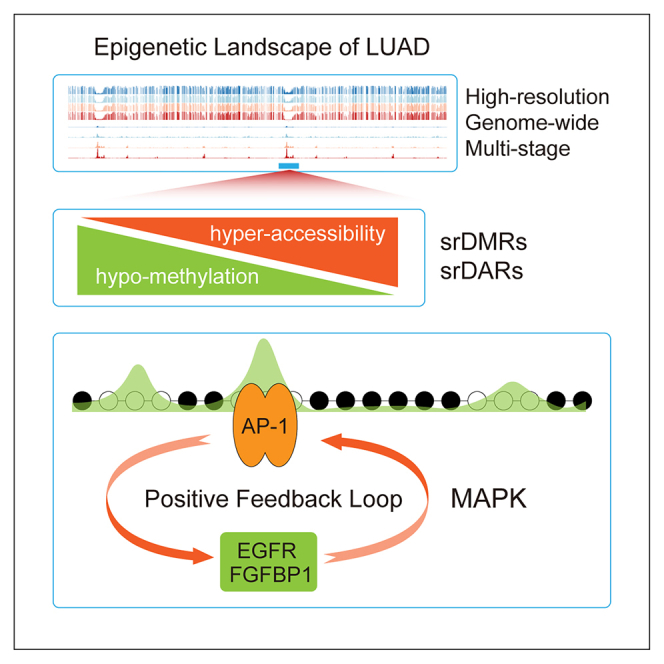

## Introduction

Lung cancer is one of the cancers with the highest global morbidity and mortality rates.^1^ Specifically, lung adenocarcinoma (LUAD) is the most prevalent histological subtype of lung cancer and has been extensively studied.^2^ LUAD is categorized into distinct stages: adenocarcinoma *in situ* (AIS), minimally invasive adenocarcinoma (MIA), and invasive adenocarcinoma (IAC),^3^ with molecular investigations revealing a sequential progression from AIS through MIA to IAC.^4^^,^^5^^,^^6^ DNA methylation, a pivotal epigenetic mechanism in mammals, involves the addition of methyl groups at the C5 position of cytosine, resulting in 5-methylcytosine formation. This modification has been observed to deviate from normal levels in various human pathologies, including cancer, underscoring its potential role in the disease’s pathogenesis.^7^^,^^8^^,^^9^ The unique DNA methylation pattern of lung cancer has been proposed as a classifier,^10^ detector,^11^ and evaluator.^12^ The DNA methylation landscape has been utilized to investigate cancer progression. In advanced prostate cancer, whole-genome methylation profiling has identified distinct hypermethylation subtypes and regulatory alterations associated with oncogene activation and tumor evolution.^13^ Similarly, in glioblastoma, DNA methylation mapping revealed spatiotemporal heterogeneity related to the cancer development and tumor microenvironment.^14^ Multiple versions of the methylation profiles of lung cancer cells have been generated for further study.^15^^,^^16^^,^^17^ Chromatin accessibility, a critical aspect of epigenetic regulation, equally significant to DNA methylation,^18^^,^^19^ provides a more accurate depiction of chromatin structure than DNA methylation. Some chromatin accessibility profiles of the human genome have been discussed.^20^^,^^21^^,^^22^ Similar to the DNA methylation landscape, chromatin accessibility profiles can also be used to study cancer progression. For example, chromatin accessibility profiles revealed ASCL1-driven epigenetic remodeling that promotes lineage plasticity and treatment resistance in prostate cancer.^23^ Additionally, chromatin accessibility analysis demonstrated that 2-hydroxyglutarate disrupts chromatin stability, leading to increased cellular heterogeneity and poor prognosis in breast cancer.^24^ Epigenetic landscapes derived from DNA methylation and chromatin accessibility have been extensively utilized in cancer research. To more comprehensively capture the epigenetic features of cancer, integrative multi-omics landscapes combining these two modalities are required. A high-resolution, genome-wide, multi-stage epigenetic landscape of LUAD, integrating both DNA methylation and chromatin accessibility, has not yet been established. To address this gap, this study employed whole-genome bisulfite sequencing (WGBS)^25^^,^^26^ and assay for transposase-accessible chromatin with sequencing (ATAC-seq)^27^ to elucidate the epigenetic attributes and transitions across different stages of LUAD.

The mitogen-activated protein kinase (MAPK) pathway is a crucial intracellular cascade that regulates cellular responses to extracellular stimuli, playing a central role in processes such as cell proliferation, differentiation, and survival.^28^ In lung cancer, the MAPK pathway manifests as an intricate network of molecular interactions, with selected targets emerging as seminal nodes that dictate disease pathogenesis.^29^ Molecules such as Raf protein kinase (RAF), extracellular regulated protein kinase (ERK), and MAPK kinase (MEK) play critical roles in choreographing a sequence of biochemical events that significantly influence cellular proliferation, survival, and differentiation.^30^^,^^31^ Recent studies in dilated cardiomyopathy (DCM) have revealed that lipidomic alterations directly modulate MAPK signaling activity, particularly through stress-induced p38 MAPK activation, which may parallel lipid-driven MAPK dysregulation in cancer progression.^32^ These molecules modulate the activity of downstream transcription factors (TFs) in the MAPK pathway, including AP-1 (a heterodimer composed of FOS and JUN), ETS1, EKL1, EKL4, and FOXO, thereby influencing vital cellular functions. Studies indicate that TFs can interact with epigenetic modifications, thereby regulating downstream gene expression.^33^^,^^34^^,^^35^ The extracellular components of the MAPK pathway include receptor tyrosine kinases (RTKs) and growth factors (GFs).^36^ EGFR, a member of the RTK family, is a well-established driver of lung cancer.^37^ Additionally, FGFBP1 enhances the signaling cascade initiated by FGF, a type of GF, within the MAPK pathway.^38^

In this study, a high-resolution, genome-wide, multi-stage epigenetic landscape of LUAD was established. Using this landscape, epigenetic biomarkers that effectively distinguish between different stages of LUAD were identified. Furthermore, using multi-omics technology, the role of epigenetics in mediating LUAD progression was investigated. The role of AP-1 family members in the MAPK cascade in LUAD has been previously elucidated. Moreover, the findings of this study demonstrate that hypo-DNA methylation and hyperchromatin accessibility at AP-1 binding sites lead to the increased expression of downstream genes, potentially activating the MAPK pathway. This creates a positive feedback loop (MAPK→AP-1/Epigenetic Modifications→EGFR/FGFBP1→MAPK) critical in lung cancer progression, particularly in IAC, offering insights into targeted therapeutic strategies.

## Results

### Genome-wide DNA methylation profiles in lung adenocarcinoma

**Figure 1:**
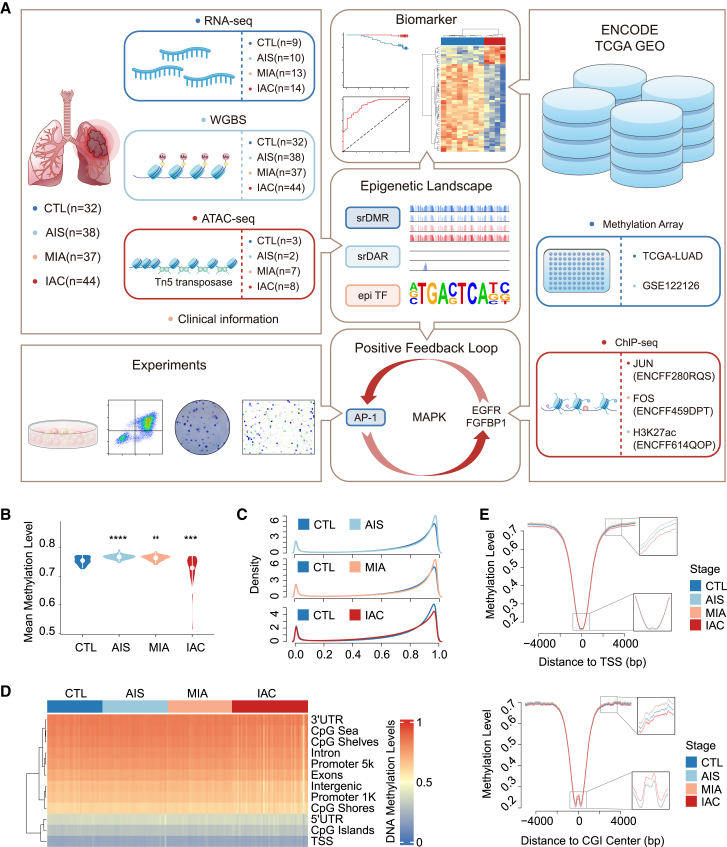
Experimental design and genome-wide DNA methylation profiles in LUAD across different stages (A) Experimental design of this article. The sharp-cornered rectangle depicts the experimental data and methods, while the rounded rectangle depicts the main result of this study. (B) Comparative analysis of the mean DNA methylation level across samples within the groups CTL, AIS, MIA, and IAC. Statistical comparisons were made using unpaired two-tailed Student’s t test (∗*p* < 0.05, ∗∗*p* < 0.01, ∗∗∗*p* < 0.001, ∗∗∗∗*p* < 0.0001). (C) Distribution of DNA methylation level densities for CTL versus AIS (top), CTL versus MIA (middle), and CTL versus IAC (bottom). (D) Evaluation of average DNA methylation levels in specific genomic regions for each group (CTL, AIS, MIA, and IAC). (E) Analysis of CpG methylation levels within a 5,000 bp range upstream and downstream of the TSS (top) and CGI (bottom) across the groups CTL, AIS, MIA, and IAC.

For this study, 151 lung tissue specimens were collected from patients and stratified into four cohorts: control conditions such as pneumonia and pulmonary fibrosis (CTL), AIS, MIA, and IAC (Figure 1A, Table S1). This analysis revealed no significant differences in age or smoking status across the groups, nor was a significant correlation between sex and cancer classification observed (Table S2). WGBS was subsequently conducted to assess the global DNA methylation landscape, achieving an average bisulfite conversion rate of 99.41% and an average CpG coverage depth of 25.8 across all specimens (Table S3). Relative to CTL, a marked increase in mean methylation levels in AIS and MIA was observed, while a significant decrease was noted in IAC (Figure 1B). The methylation profiles for each cohort were synthesized to represent CTL, AIS, MIA, and IAC, which mirrored the sample-specific average DNA methylation findings. Specifically, the methylation density distribution across the stages indicated a methylation gain in AIS and MIA, contrasting with a loss in IAC (Figure 1C). Furthermore, analysis of methylation patterns across specific genomic regions (Figure 1D) revealed that transcription start sites (TSS) exhibited the lowest average methylation, whereas the 3′ untranslated regions (3′UTRs) showed the highest. Methylation levels increased with distance from CpG islands (CGIs). Notably, IAC samples demonstrated a higher coefficient of variation in methylation levels than the other groups (Table S4). Hypomethylated regions were predominantly located near the TSS and CGI centers across all cohorts (Figure 1E), with IAC stages exhibiting uniquely higher methylation at the CGI centers than at the other stages.

### Alterations in DNA methylation during the progression of lung adenocarcinoma

**Figure 2:**
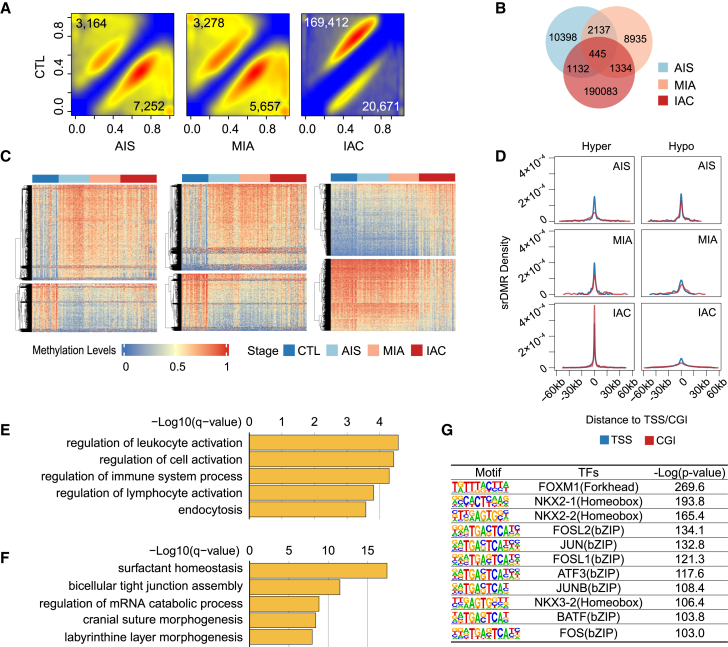
Differential methylation in LUAD across different stages (A) Distribution of DMC density comparing CTL with AIS (left), CTL with MIA (middle), and CTL with IAC (right). The numbers in the upper left and lower right corners indicate the number of hypo-DMCs and hyper-DMCs, respectively. (B) Venn diagram of DMCs identified in each stage. (C) Heatmaps depicting srDMCs for AIS (left), MIA (middle), and IAC (right). The top section of each heatmap illustrates hyper-srDMCs, while the bottom section shows hypo-srDMCs. (D) Density plots illustrating the distribution of srDMRs in proximity to the TSS (blue) and CGIs (red). (E and F) Results from GREAT analysis of hyper-srDMRs in AIS (E) and hypo-srDMRs in IAC (F). (G) TFs significantly enriched in hypo-srDMRs in IAC obtained by HOMER motif analysis.

Differentially methylated CpGs (DMCs) were identified in this genome-wide analysis by comparing the AIS, MIA, and IAC stages with the CTL group, encompassing 29,401,360 CpGs (Tables S5A–S5C). In AIS, 7,252 of CpGs were hypermethylated (hyper-DMCs) and 3,164 were hypomethylated (hypo-DMCs). These proportions shifted to 5,657 hyper-DMCs and 3,278 hypo-DMCs in MIA and markedly to 20,671 and 169,412 in IAC, respectively (Figures 2A and 2B). This trend indicated a progressive increase in DMCs from AIS to IAC, with a notable tendency toward hypomethylation as LUAD advanced (Table S5D). In contrast, specific genomic regions exhibited an increase in hyper-DMCs from AIS to IAC, particularly within CGIs (Table S6).

By analyzing DMC alterations across the stages, DMCs associated with LUAD progression (stage-related DMCs [srDMCs]) were identified (Tables S7A–S7C). The srDMCs were classified into six categories based on their initial stage of appearance: hyper-DMCs and hypo-DMCs in AIS, MIA, and IAC (Figure 2C, Table S7D). Additionally, 15,491 stage-related differentially methylated regions (srDMRs) based on srDMCs that were predominantly concentrated near the TSS and CGIs were identified (Table S8, Figure 2D). Notably, the density of hyper-DMRs near CGIs in IAC was higher than that in TSS. The genomic regions enrichment of annotations tool (GREAT) analysis^39^^,^^40^ of these srDMRs revealed that hyper-DMRs in AIS were linked to immune-related biological processes (Figure 2E). In contrast, hypo-DMRs in IAC regulated processes such as surfactant homeostasis, bicellular tight junction assembly, and other immune, morphogenic, and metabolic processes (Figure 2F). Surfactant homeostasis refers to the intricate regulation of lung surfactants by alveolar type II cells, which are essential for pulmonary function and preventing alveolar collapse.^41^

Longer srDMRs were predominantly found in IAC (Figure S1A), indicating deterioration in lung cancer. Using HOMER,^42^ 94 TFs enriched in these srDMRs were identified (Figures S1B and S1C). The AP-1 family, with a leucine zipper (bZIP) domain, exhibited significant enrichment among the hypo-srDMRs in IAC (Figure 2G). AP-1 was also identified within the hyper-srDMRs in AIS. Previous studies have implicated AP-1 in cell migration and invasion.^43^^,^^44^

### Chromatin accessibility landscape highlights the enrichment of transcription factor binding sites

**Figure 3:**
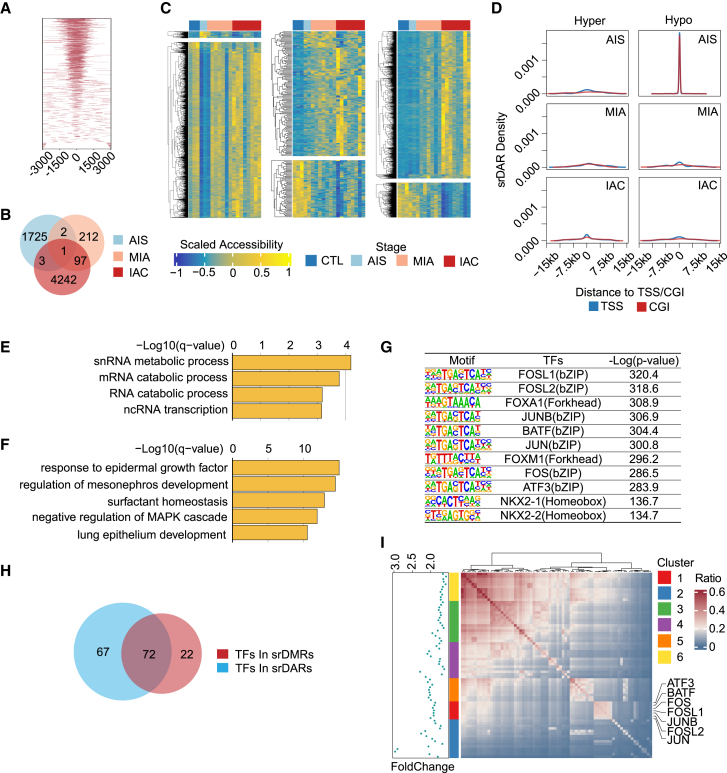
Chromatin accessibility landscape and epi TFs in LUAD progression (A) Distribution of ATAC-seq peaks across stages CTL, AIS, MIA, and IAC. (B) Venn diagram of DARs identified in each stage. (C) Heatmaps displaying srDARs for AIS (left), MIA (middle), and IAC (right). (D) Density plots illustrating the distribution of srDARs relative to the TSS (blue) and CGIs (red). (E and F) GREAT analysis of hypo-srDARs in AIS (E) and hyper-srDARs in IAC (F). (G) TFs significantly enriched in hyper srDARs in IAC obtained by HOMER motif analysis. (H) The common epi TFs enriched in both srDMRs and srDARs by HOMER. (I) Classification of 72 epi TFs into 6 groups based on co-localization ratios within srDARs. The heatmap indicates the proportion of shared binding sites between TF pairs, with the adjacent histogram depicting the ratio change of shared sites in srDARs relative to the whole genome.

To elucidate the chromatin accessibility landscape in different LUAD subtypes, ATAC-seq was conducted on 20 samples (Table S9) from the CTL, AIS, MIA, and IAC groups, and 189,351 accessibility peaks were identified (Figure 3A). Differential chromatin accessibility regions (DARs) were identified across the three LUAD stages (Figure 3B). Similar to srDMRs, these stage-related DARs (srDARs) were categorized into six groups based on their initial stage of emergence: 37 hyper-accessibility DARs (hyper-DARs) and 1,688 hypo-accessibility DARs (hypo-DARs) in AIS, 144 hyper-DARs and 66 hypo-DARs in MIA, and 3,907 hyper-DARs and 236 hypo-DARs in IAC (Tables S10A–S10C, Figure 3C). Notably, hypo-DARs in AIS exhibited a significantly higher density near the TSS and CGIs than other srDARs (Figure 3D), indicating their involvement in snRNA metabolic processes (Figure 3E), which are crucial for immune system regulation, gene expression, and RNA splicing. In contrast, hyper-DARs in IAC were linked to biological processes associated with epidermal growth factor (EGF), MAPK, and surfactant homeostasis (Figure 3F).

The DNA-binding sites of 139 TFs were enriched in the srDARs across the AIS, MIA, and IAC stages of LUAD (Figures S1A–S1C). The AP-1, FOX (FOXA1 and FOXM1), and HOX (NKX2-1 and NKX2-2) families showed the most significant enrichment among the hyper-srDARs in IAC (Figure 3G). A total of 72 TFs, including these TFs, were identified in both srDMRs and srDARs (Figure 3H). Epigenetic modifications at each stage can influence LUAD progression via these TFs. The DARs containing the binding sites of these 72 common TFs were analyzed, and these TFs were organized into six clusters based on their colocalization within the same DARs (Figures 3I and S2A). We named these epigenetic-related TFs (epi TFs) in LUAD. Cluster 1 in epi TFs comprised the AP-1 family, with members such as FOS and JUN, which are frequently dysregulated in cancer, promoting unchecked cell growth and tumorigenesis.

### Gene expression profile alterations throughout the progression of lung adenocarcinoma

**Figure 4:**
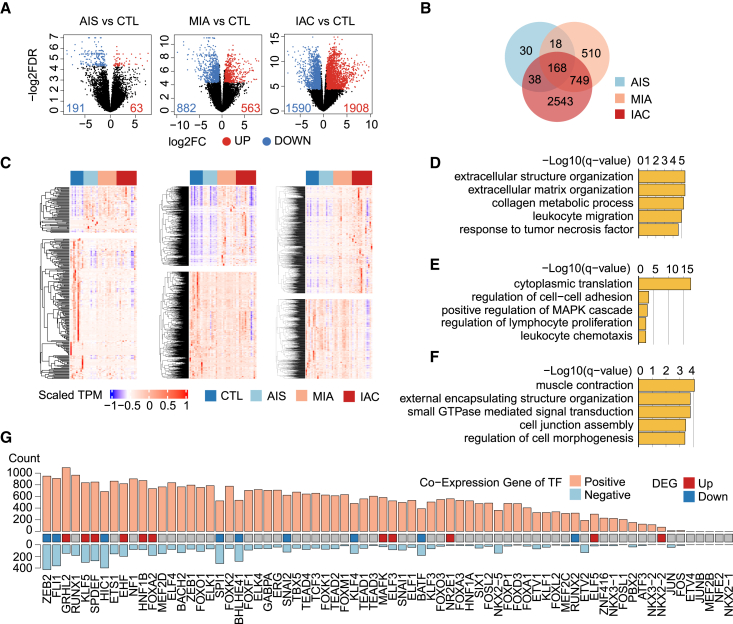
Gene expression profile and functional analysis of LUAD (A) DEGs across stages AIS, MIA, and IAC. (B) Venn diagram of DEGs identified in each stage. (C) Heatmaps delineating srDEGs for AIS (left), MIA (middle), and IAC (right). (D–F) GO enrichment analyses of srDEGs for AIS (D), MIA (E), and IAC (F). (G) Count of genes co-expressed with epi TFs. Red: upregulated TFs, blue: down-regulated TFs.

To elucidate the epigenetic dynamics across LUAD stages, expression profiles were analyzed by selecting 46 tissue samples for simultaneous WGBS and RNA sequencing (RNA-seq) (Table S11). This analysis revealed the following stage-specific differentially expressed genes (DEGs): 254 in AIS, 1,445 in MIA, and 3,498 in IAC (Figure 4A, Table S12). Notably, most DEGs identified in AIS and MIA were also present in IAC, with a significant number uniquely emerging at the advanced stage (Figure 4B). These DEGs, similar to srDMRs and srDARs, were further categorized as stage-related DEGs (srDEGs) (Figure 4C). Specifically, the initial emergence of 254 DEGs in AIS was linked to immune processes and aligned with early hypermethylation domains and chromatin accessibility changes (Figure 4D). DEGs in MIA were associated with the MAPK cascade, as evidenced by hyper-DARs in IAC (Figure 4E). Additionally, 2,543 DEGs that first appeared in IAC were enriched for small GTPase signal transduction related to the MAPK pathway (Figure 4F).

To investigate the co-expression patterns between epi TFs and DEGs, Pearson correlation coefficients were calculated for each TF-DEG pair across all samples (CTL, AIS, MIA, and IAC). A TF and DEG pair was considered co-expressed when *p*-value <0.05. Pairs with *r* > 0 were classified as positively correlated, and those with *r* < 0 as negatively correlated. Certain TFs exhibited a high number of co-expressed genes, while others demonstrated a limited number of co-expressed genes (Figure 4G). Differentially expressed TFs tended to have more co-expressed genes than other TFs, with only 20 epi TFs demonstrating significant differential expression across LUAD stages (Figure S2B). Although some epi TFs, such as members of the AP-1 family (e.g., JUN and FOS), were not differentially expressed in our dataset and showed only a limited number of co-expressed target genes (Figure S2C), their well-established roles as key transcriptional regulators suggest that epi TFs may still influence gene expression through additional mechanisms beyond expression level alone. For example, previous studies have demonstrated that AP-1 regulates downstream targets through transcriptional activation^45^ and post-translational^46^ mechanisms, the influence of epigenetic on AP-1 activity remains to be elucidated.

### Chromatin accessibility is negatively correlated with DNA methylation levels in hyper-differential chromatin accessibility regions of invasive adenocarcinoma

**Figure 5:**
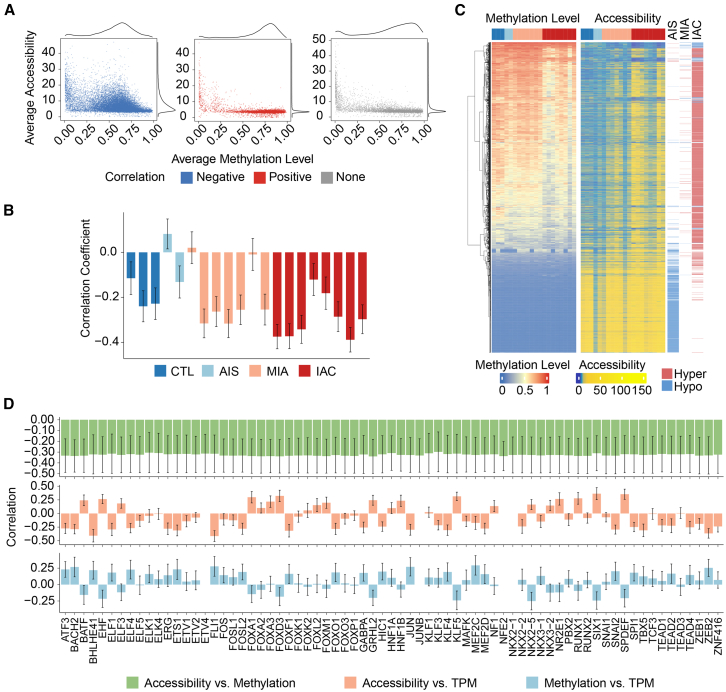
Relationship between chromatin accessibility and DNA methylation levels in LUAD (A) Average methylation and average accessibility of ATAC-seq peaks whose methylation levels are negatively (left), positively (middle) and not (left) correlated with accessibility. (B) Correlation (mean ± SD) between methylation rate and chromatin openness across samples in groups AIS, MIA, and IAC. (C) Heatmap visualization of the interrelation between DNA methylation levels and chromatin accessibility across DARs. (D) Multi-dimensional correlation analysis at the binding sites of 72 epi TFs: methylation levels versus chromatin accessibility (top), chromatin accessibility versus TF expression (middle), and methylation levels versus TF expression (bottom). Correlations are presented as mean ± SD.

The above investigations demonstrated the enrichment of epi TFs in both srDMRs and srDARs, suggesting a potential association between methylation and chromatin accessibility in these regions. Therefore, the interplay between DNA methylation levels and chromatin accessibility was further explored, and their combined impact on the initial stages of lung cancer progression was assessed. By extracting methylation levels from ATAC-seq peak regions, a Kendall correlation analysis was conducted between peak accessibility and methylation levels. In total, 15,411 regions exhibited a positive correlation between accessibility and methylation levels, whereas 5,977 regions showed a negative correlation. Regions with negative correlations tended to exhibit increased accessibility and reduced methylation levels (Figure 5A).

To study differences in the correlation between chromatin accessibility and methylation levels across different groups, the correlation within individual samples was estimated through sampling. A decrease in the correlation coefficient with LUAD progression was observed (Figure 5B). Notably, most DARs did not significantly correlate with methylation levels, except for hyper-DARs in the IAC stages, where accessibility was inversely correlated with methylation levels (Table S13). A comparative analysis of DNA methylation and chromatin accessibility within srDARs indicated that hypo-DARs in IAC were primarily located in highly methylated regions (Figure 5C). Cumulatively, these findings suggest a negative correlation between methylation levels and chromatin accessibility in hyper-DARs in IAC, with most epi TFs preferentially binding to these regions (Figures 5D and S2A).

### Methylation patterns of stage-related differential chromatin accessibility regions as predictive biomarkers for lung cancer

**Figure 6:**
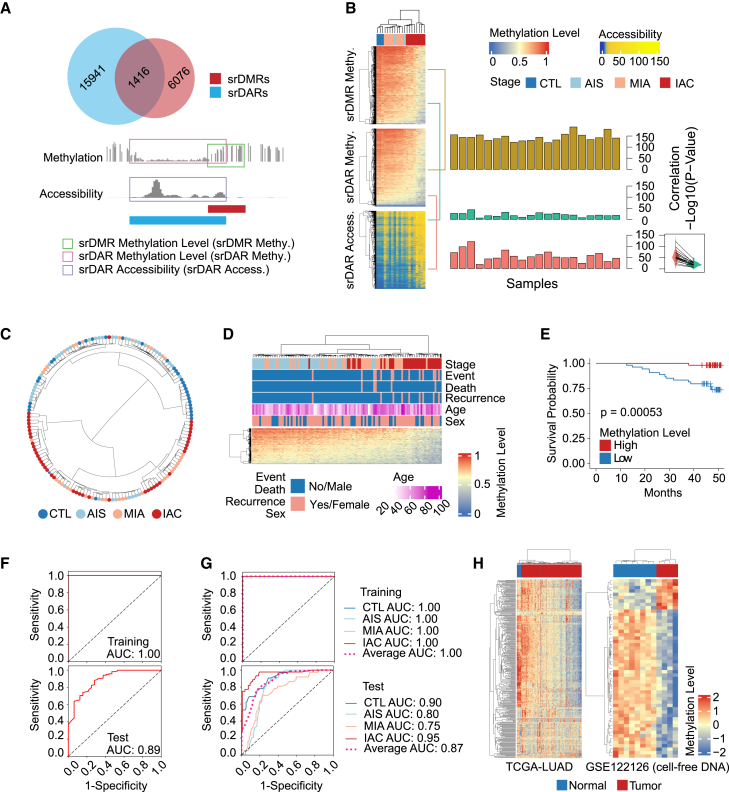
Diagnostic and prognostic potential of srDARs and srDMRs as lung cancer biomarkers (A) The 1,416 srDARs that overlap with srDMRs. Three metrics can be used to evaluate the intersection region. srDMR methylation: the methylation level of the srDMR. srDAR methylation: the DNA methylation level of the srDAR. srDAR accessibility: the chromatin accessibility of the srDAR. (B) Heatmaps displaying srDMR methylation (top), srDAR methylation (middle), and srDAR accessibility (bottom). The bar plot represents the -log10(*p*-value) of Pearson correlation coefficients between srDMR methylation and srDAR methylation (top), srDMR methylation and srDAR accessibility (middle), and srDAR methylation and srDAR accessibility (bottom) of each sample. Violin diagrams represent the -log10(*p*-value) of Pearson correlation coefficients between srDAR methylation and srDAR accessibility (left) and srDMR methylation and srDAR accessibility (right). (C) Hierarchical clustering based on DNA methylation levels of 1,416 srDARs. (D) Comprehensive heatmap of the 1,416 srDARs, annotated with clinical data including patient survival, recurrence, age, and gender. Event denotes either death or recurrence. (E) Survival analysis curves comparing average methylation levels of 1,416 srDARs. (F) Receiver operating characteristic (ROC) curve for a binary classification model discerning cancerous from non-cancerous tissues, presented for both training (top) and test (bottom) datasets. (G) ROC curve for a multi-class model distinguishing between CTL, AIS, MIA, and IAC stages, with performance metrics for training (top) and test (bottom) datasets. (H) Heatmap of CpG methylation levels in 1,416 srDARs within the lung tissue (TCGA-LUAD, left) and cell-free DNA (GEO: GSE122126, right) datasets.

As alterations in the epigenetic landscape can influence the transcriptional activity of TFs, whether the 1,416 regions with significantly altered methylation levels and chromatin accessibility (Figure 6A) were associated with the clinical manifestations of lung cancer was investigated. These regions can be quantitatively represented by three metrics: srDMR methylation level, srDAR methylation level, and srDAR accessibility. A statistically significant correlation was observed among these metrics, particularly between srDAR and srDMR methylation levels (Figure 6B). Furthermore, srDAR methylation levels exhibited a stronger correlation with srDAR accessibility than srDMR methylation levels. Consequently, the srDAR methylation level was selected as a key indicator for assessing the epigenetic characteristics of these 1,416 regions. Most patient samples were accurately classified based on these regions (Figure 6C). Lower methylation levels in these 1,416 regions were associated with increased recurrence and mortality (Figure 6D), suggesting a poorer prognosis (Figure 6E). To explore the potential of these regions as biomarkers, a random forest model was used to distinguish LUAD from non-cancerous samples, achieving a high area under the curve (AUC) of 0.89 in the test set (Figure 6F). Additionally, a multi-classification model further differentiated the CTL, AIS, MIA, and IAC stages with an average AUC of 0.87 in the test set. Notably, the AUC for CTL and IAC reached 0.90 and 0.95, respectively (Figure 6G), highlighting the model’s efficacy in classifying early and advanced disease stages.

The TCGA-LUAD dataset^47^ was used to verify the srDARs (Figure S3A). By selecting sites within these 1,416 regions for modeling (Figure 6H), a distinction was made between cancerous and paracancerous tissues. The AUC of both the training and test sets was 1.0 (Figure S3B). Additionally, methylation data from cell-free DNA (GEO: GSE122126) was used to confirm the presence of these abnormally methylated regions in serum. The sites within these srDARs could distinguish between cancerous serum samples and those from healthy individuals, emphasizing the potential of the methylation levels of these 1,416 regions as effective clinical biomarkers (Figure 6H). These assessments affirm the clinical relevance of srDAR methylation levels, offering a promising avenue for diagnosis and prognosis and potentially influencing treatment strategies in lung cancer, thereby advancing the application of epigenetic biomarkers in clinical settings.

### Positive feedback loop involving mitogen-activated protein kinase and epigenetic regulation in lung adenocarcinoma

**Figure 7:**
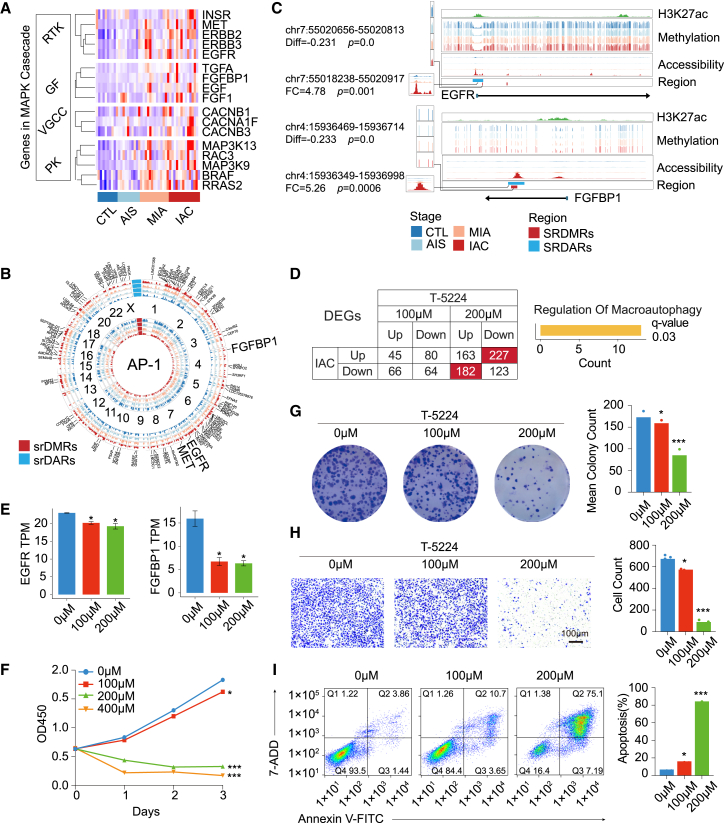
Interplay of MAPK signaling and epigenetic regulation in LUAD (A) Highly expressed genes in the MAPK pathway in LUAD. RTK, receptor tyrosine kinase; GF, growth factor; VGCC, voltage-gated calcium channel. PK: protein kinases. (B) DEGs regulated by AP-1 in srDARs. The inner four-layer bar plot illustrates the methylation level at each stage, the outer four-layer bar plot depicts chromatin accessibility across these stages, and the outermost layer represents the DEGs regulated by AP-1. (C) Methylation levels and chromatin accessibility profiles near EGFR (top) and FGFBP1 (bottom) regulatory regions. Green peaks indicate regions of active transcription at the promoters or enhancers. (D) DEG count of stage IAC and A549 cells (left) and GO enrichment analyses of genes in the red grid (right). 100 μM: A549 treatment with 100 μM T-5224; 200 μM: A549 treatment with 200 μM T-5224; Up: up-regulated genes; Down: down-regulated genes. (E) Expression level (mean ± SD, *n* = 3) of EGFR (left) and FGFBP1 (right) of A549 across a range of T-5224 concentrations (0 μM, 100 μM, and 200 μM). Unpaired two-tailed t-tests were used (∗*p* < 0.05, ∗∗*p* < 0.01, ∗∗∗*p* < 0.001, ∗∗∗∗*p* < 0.0001). (F) Quantitative assessment of A549 cell viability using a CCK-8 assay across a range of T-5224 concentrations (0 μM, 100 μM, 200 μM, and 400 μM). Paired two-tailed t-tests were used (∗*p* < 0.05, ∗∗*p* < 0.01, ∗∗∗*p* < 0.001, ∗∗∗∗*p* < 0.0001). (G–I) Colony-formation assay (G), transwell migration assay (H), and flow cytometry apoptosis assays (I) of A549 under various concentrations of T-5224 treatment (0 μM, 100 μM, and 200 μM). Scale bar in (H): 100 μm. *n* = 3 biological replicates per condition. Unpaired two-tailed t-tests were used (∗*p* < 0.05, ∗∗*p* < 0.01, ∗∗∗*p* < 0.001, ∗∗∗∗*p* < 0.0001).

Enrichment analysis of srDARs and srDEGs revealed a significant association between LUAD and the MAPK pathway (Figure S4A). Alterations in the expression of key members of the MAPK cascade as lung cancer progressed were identified in this study. Upregulated genes in the MAPK pathway, including RTKs, GFs, voltage-gated calcium channels (VGCCs), and protein kinases (PKs), were observed (Figure 7A). These genes enhance cellular responsiveness to extracellular signals, which increase MAPK pathway activity. Specific epi TFs, namely AP-1 (JUN, FOS), ELK1, ELK4, ETS1, and FOXO3, were identified in the terminal phase of the MAPK pathway. Upregulation of MAPK pathway downstream genes, including MMP3^48^ and MMP7,^49^ indicated increased activation of the MAPK cascade in LUAD (Figure S4B). The above research results underscore the AP-1 family as a set of epi TFs significantly enriched in both srDMRs and srDARs. Taken together, the evidence suggests that AP-1 may act as a crucial intermediary between MAPK signaling and epigenetic modulation in LUAD, thereby justifying its central role in the present study.

Motif analysis of ATAC-seq peaks revealed 5,740 binding sites of AP-1 within srDARs. These binding sites were further filtered using chromatin immunoprecipitation sequencing (ChIP-seq) data for JUN (ENCODE: ENCFF280RQS), FOS (ENCODE: ENCFF459DPT), and H3K27ac (ENCODE: ENCFF614QOP) in A549 cells, providing experimental evidence that AP-1 binds to regions with significant transcriptional activity. Finally, the binding of AP-1 to 819 srDARs in LUAD was verified. These AP-1 target binding sites reside within or near the transcriptional regulatory regions of 114 DEGs, including critical MAPK cascade activators such as EGFR, FGFBP1, and MET (Figure 7B). Significant alterations in methylation levels and chromatin accessibility near the TSS of these genes were identified (Figures 7C and S4C). The expression levels of EGFR, FGFBP1, and MET showed a general trend of positive correlation with chromatin accessibility and negative correlation with the methylation levels of their regulatory regions containing AP-1 binding sites. However, some of these correlations were weak or did not reach statistical significance, possibly due to limited sample size or the presence of outliers (Figure S4D).

To study the regulatory effect of AP-1 binding on downstream genes, A549 cells were treated with the AP-1 inhibitor T-5224. A concurrent increase in DEGs with increasing inhibitor concentrations was observed (Figure S4E, Table S13). T-5224 counteracted the abnormal expression of genes involved in the regulation of macroautophagy in IAC (Figure 7D); autophagy can induce the apoptosis and migration of cancer cells.^50^ Among the 114 target genes of AP-1 identified in LUAD tissues, 59 were differentially expressed in the A549 cell line (Figure S4F). Notably, the expression levels of EGFR and FGFBP1 decreased upon inhibition of AP-1 binding (Figure 7E), with FGFBP1 significantly associated with survival in patients with LUAD (Figures S4G and S4H).^51^ The reduced expression levels of MMP3 and MMP7 indicated that T-5224 inhibited MAPK pathway activity (Figure S4I). This finding reveals a positive feedback pathway involving MAPK, AP-1, and epigenetic regulation, contributing to the modulation of the MAPK signaling cascade. This feedback loop further augmented the expression of downstream AP-1 target genes, including EFNA5 and PERP, thereby driving lung cancer progression (Figures S4J and S4K).

As a key hub in this positive feedback loop, the function of AP-1 in lung cancer was studied in A549 cells treated with different concentrations of T-5224. Treatment with T-5224 resulted in a significant and dose-dependent reduction in A549 cell proliferation (Figure 7F), colony formation (Figure 7G), and cell migration (Figure 7H). Moreover, T-5224 exhibited a dose-dependent effect on the induction of apoptosis in A549 cells, as evidenced by an increase in the apoptosis rate from 5.30% in control cells to 14.35% and 82.29% at concentrations of 100 μM and 200 μM, respectively (Figure 7I). In conclusion, these findings suggest that the TF AP-1 is a potential therapeutic target in LUAD.

## Discussion

AIS and MIA are early stages of LUAD, typically characterized by favorable post-treatment prognoses. In contrast, patients diagnosed with IAC have a less favorable prognosis.^3^^,^^52^ Consequently, a substantial cohort of LUAD samples across diverse stages was amassed to elucidate lung cancer progression through an epigenetic lens. The WGBS and ATAC-seq data revealed a high-resolution, genome-wide, and multi-stage epigenetic landscape of LUAD. In the AIS and MIA stages, the DNA methylation level and chromatin accessibility exhibited minor alterations, contrasting with significant changes in IAC. This provides epigenetic evidence for LUAD evolution from MIA to IAC.^4^^,^^5^^,^^6^^,^^53^ Subsequently, srDMRs and srDARs enriched in gene loci associated with critical biological processes were identified, including MAPK signaling, immune regulation, cell adhesion, proliferation, differentiation, and migration. The 1,416 common regions were selected for further study. Strikingly, the results revealed a significant association between hypomethylation in these distinct regions and poor LUAD prognosis. Modeling these regions using a random forest model enabled the differentiation of different LUAD stages. Some methylation signals in these intervals have been found in serum, and further research will be conducted to develop panels for liquid biopsy with high sensitivity and specificity.^54^

Epigenetic modifications and TF binding mutually influence each other.^55^^,^^56^^,^^57^ In total, 72 epi TFs in epigenetically altered LUAD regions were identified and divided into six clusters based on shared binding sites. Cluster 1 included TFs such as FOS and JUN, with binding sites more likely to appear in srDMRs and srDARs. Since AP-1 is a member of the MAPK pathway, this explains the enrichment of MAPK in the hyper-DARs observed in IAC. Combining experimental proofs with existing studies reporting the function of AP-1 in cancer,^43^^,^^58^^,^^59^^,^^60^ the assertion can be made that AP-1 and its related epigenetic mechanisms play a crucial role in LUAD progression. Other non-epi TFs may contribute to the progression of LUAD by interacting with epi TFs. For example, E2F2 has been shown to promote LUAD progression through a core transcriptional regulatory circuitry facilitated by B-Myb and FOXM1.^61^ In addition, GATA6 and HOPX have been implicated in the regulation of LUAD metastasis, exhibiting complex cooperative and regulatory relationships with NKX2-1.^62^

Using RNA-Seq data, increasing transcriptional dysregulation of genes associated with LUAD was observed. This, together with DNA methylation levels and chromatin accessibility, reveals the molecular evolution from AIS to IAC during lung cancer development. Some members of the MAPK pathway are aberrantly expressed in LUAD, with Gene Ontology (GO) enrichment analysis of DEGs in IAC highlighting the role of the MAPK pathway in lung cancer progression. Key elements of the MAPK pathway, such as EGFR, RAS, ERK, and MET, are recognized lung cancer therapeutic targets. However, drug resistance often occurs during targeted drug treatments.^63^ As a downstream hub of the MAPK pathway, AP-1 can prevent abnormal signals from the upstream bypass pathway^30^^,^^64^ and is a potential drug target.

Specific downstream genes, such as EGFR, FGFBP1, and MET, capable of activating the MAPK pathway through AP-1 binding sites were identified in this study, which also showed that AP-1 binds to the transcriptional regulatory regions of EGFR and FGFBP1 and regulates their transcription levels. Methylation levels and chromatin accessibility at AP-1 binding sites correlated with EGFR and FGFBP1 transcription levels, indicating that epigenetic features and AP-1 jointly affect downstream gene expression. This finding delineates an epigenetics-mediated positive feedback loop linking MAPK to AP-1 and vice versa, reinforcing the MAPK pathway. Within this circuit, epigenetic mechanisms play a central role in modulating the expression of downstream genes via AP-1. Numerous other TFs are near AP-1 binding sites in srDMRs and srDARs that can interact with factors such as DNA methylation and chromatin accessibility.^55^^,^^56^^,^^57^ Similar to other systems, positive feedback from cells tends to cause instability, and this epigenetics-mediated loop disrupts intracellular transcriptional harmony, leading to LUAD deterioration.

In conclusion, the epigenetic landscape elucidated across different stages of LUAD in this study holds significant implications for advancing various avenues of research. While the epigenetic-mediated positive feedback loop requires further empirical investigation, the framework outlined herein offers fundamental insights into epigenetic modulation within LUAD, setting the stage for more detailed future research.

### Limitations of the study

Utilizing the epigenetic landscape, lung cancer was investigated from two perspectives: molecular markers and functional mechanisms. However, the present study was subject to certain limitations. First, this investigation yielded improved biomarkers and models exclusive to lung cancer tissue that may not be directly applicable or generalizable to liquid biopsy. Moving forward, this issue will be addressed by collecting blood samples from multiple centers and utilizing WGBS data of cell-free DNA from these samples to further explore the 1,416 regions exhibiting significant epigenetic changes highlighted in this study. Additionally, while the AP-1 family comprises multiple members, this study did not distinguish between individual members. To elucidate the specific involvement of each member in the epigenetic-mediated positive feedback loop observed in LUAD, further investigations will include more intricate and targeted experimental designs.

## Resource availability

### Lead contact

Further information and requests for resources, reagents, and data should be directed to and will be fulfilled by the lead contact, Deqiang Sun (deqiangs@zju.edu.cn).

### Materials availability

This study did not generate unique reagents.

### Data and code availability

• The intermediate analysis files for WGBS, ATAC-seq, and RNA-seq reported in this article have been deposited (BioProject: PRJCA024580) in the Genome Sequence Archive of the BIG Data Center (https://ngdc.cncb.ac.cn/omix/). ChIP-seq data for JUN (ENCODE: ENCFF280RQS), FOS (ENCODE: ENCFF459DPT), and H3K27ac (ENCODE: ENCFF614QOP) were accessed using Encode (https://www.encodeproject.org/). The methylation array of LUAD tissues was obtained from TCGA-LUAD (https://portal.gdc.cancer.gov/projects/tcga-luad). Cell-free DNA methylation arrays from healthy individuals and patients with lung cancer (GEO: GSE122126) were obtained from the GEO database (https://www.ncbi.nlm.nih.gov/geo).
• The associated accession codes of this article are available on GitHub (https://github.com/hcyvan/epiLUAD).
• Any additional information required to reanalyze the data reported in this article is available from the lead contact upon request.

## Acknowledgments

We gratefully acknowledge the financial support provided by the 10.13039/501100001809National Natural Science Foundation of China (NSFC, Project 81773012) and the 10.13039/501100012166National Key Research and Development Program of China (2022YFA1105200 and 2023YFA1800700).

## Author contributions

All authors assisted in conducting the study. D.S., Z.C, Y.T., Y.B., and B.Z. participated in the conception and design; Y.C., Y.L., T.H., and Q.R. participated in the data analysis; L.X., T.Y., Y.T., W.L., X.W, and Z.S. participated in the acquisition and preparation of specimens; Y.S., M.C., and Y.C. participated in drafting the article or revised the content of the article critically; Y.C., Y.L., and T.H. participated in reviewing the final version for publication. All authors agree to be responsible for all aspects of the work.

## Declaration of interests

The authors declare that no competing interests exists.

## STAR★Methods

### Key resources table

**Table:** 

REAGENT or RESOURCE	SOURCE	IDENTIFIER
**Biological samples**		

Lung tissues from patients	Affiliated Yixing Hospital of Jiangsu University	NA

**Critical commercial assays**		

DNeasy Blood & Tissue Kit	Qiagen	NA
EZ DNA Methylation-Gold Kit	Zymo Research	NA
Nextera DNA Library Preparation Kit	Illumina	NA
MinElute PCR Purification Kit	Qiagen	NA
KAPA Real-Time Library Amplification Kit	Roche	NA
PCRClean DX beads	Aline Biosciences	NA
AHTS Universal V8 RNA-seq Library Prep Kit for Illumina	Vazyme	NA
Cell Counting Kit-8 (CCK-8)	Dojindo	NA
c-Fos/AP-1 inhibitor T-5224	MedChemExpress	NA
Annexin V-FITC/7-AAD Apoptosis Detection Kit	Yeasen Biotechnology	NA

**Deposited data**		

Intermediate analysis files for WGBS, ATAC-seq, and RNA-seq	Genome Sequence Archive of the BIG Data Center (https://ngdc.cncb.ac.cn/omix/)	PRJCA024580
ChIP-seq data for JUN	ENCODE (https://www.encodeproject.org/)	ENCFF280RQS
ChIP-seq data for FOS	ENCODE (https://www.encodeproject.org/)	ENCFF459DPT
ChIP-seq data for H3K27ac	ENCODE (https://www.encodeproject.org/)	ENCFF614QOP
methylation array of LUAD	the TCGA data portal (https://portal.gdc.cancer.gov/)	TCGA-LUAD
methylation array of cell-free DNA	GEO (https://www.ncbi.nlm.nih.gov/geo)	GSE122126

**Experimental models: Cell lines**		

A549	Wuhan, China	NA

**Software and algorithms**		

MOABS (1.3.2)	Sun et al.^65^	https://github.com/sunnyisgalaxy/moabs
FastQC (0.11.9)	Bioinformatics tools	https://github.com/s-andrews/FastQC
fastp (0.20.0)	Chen et al.^66^	https://github.com/OpenGene/fastp
HISAT2 (2.2.1)	Kim et al.^67^	https://daehwankimlab.github.io/hisat2/
ENCODE ATAC-seq pipeline (2.12)	WDL pipeline	https://github.com/ENCODE-DCC/atac-seq-pipeline
R software (4.3.2)	the R Core Team and the R Foundation for Statistical Computing	https://www.r-project.org/
ComplexHeatmap (2.18.0)	R package	https://www.bioconductor.org/packages/release/bioc/html/ComplexHeatmap.html
ggplot2 (3.5.1)	R package	https://cran.r-project.org/web/packages/ggplot2/index.html
dplyr (1.1.3)	R package	https://cran.r-project.org/web/packages/dplyr/index.html
reshape2 (1.4.4)	R package	https://cran.r-project.org/web/packages/reshape2/index.html
edgeR (4.0.16)	R package	https://bioconductor.org/packages/release/bioc/html/edgeR.html
ChIPseeker (1.38.0)	R package	https://bioconductor.org/packages/release/bioc/html/ChIPseeker.html
DESeq2 (1.42.1)	R package	https://bioconductor.org/packages/release/bioc/html/DESeq2.html
epiLUAD	GitHub code repository containing implementations in R and Python	https://github.com/hcyvan/epiLUAD

### Experimental model and study participant details

#### Ethics approval and consent to participate

This study was approved by the Medical Ethics Committee of Yixing People’s Hospital (Approval ID 2021-040). Informed written consent was obtained from all participants.

#### Cell line

The human lung cancer cell line A549 was obtained from Procell (Wuhan, China).

### Method details

#### Patients and sample acquisition

Clinical data and samples were systematically collected from 151 patients with lung diseases (comprising 38 CTL, 33 AIS, 37 MIA, and 43 IAC cases) at the Affiliated Yixing Hospital of Jiangsu University. Haematoxylin and eosin (H&E) slides of each case were reviewed independently by two lung cancer pathologists to confirm the diagnosis. The study protocol and any subsequent amendments were approved by the Institutional Review Board. All participating patients provided written informed consent before inclusion in the study.

#### WGBS library preparation and data analysis

Genomic DNA was extracted using the DNeasy Blood & Tissue Kit (Qiagen, Valencia, CA) for library preparation. Purified DNA and 1% unmethylated lambda DNA (Promega, Madison, WI, USA) were sonicated to yield fragments ranging from 100 to 700 bp. The DNA fragments were subjected to sodium bisulfite conversion using an EZ DNA 318 Methylation-Gold Kit (Zymo Research, Orange, CA). Subsequently, the library was sequenced using an Illumina HiSeq6000.

MOABS^65^ (v1.3.2) was used to process raw WGBS data. The Mcall module was used to calculate the methylation level, and the comp module was used to merge the methylation level files of each sample and identify DMCs (–minCredibleDif = 0.20) The comp module applies a beta-binomial model to assess statistical significance. DMRs were identified with a minimum of three DMCs, and the maximum distance between two DMCs was 200 bp (script/dmc2dmr.py).

#### ATAC-seq library preparation and data analysis

Samples were enzymatically dissociated and lysed in lysis buffer. DNA was simultaneously fragmented and tagged with sequencing adapters using a Nextera DNA Library Preparation Kit (Illumina, California, USA). DNA fragments were purified and amplified using the MinElute PCR Purification Kit (Qiagen, Valencia, CA) and the KAPA Real-Time Library Amplification Kit (Roche, Basel, Switzerland), respectively. Subsequently, sequencing libraries were purified using PCRClean DX beads (Aline Biosciences, Waltham, USA). The library was then sequenced on NextSeq 500 (Illumina). The ENCODE ATAC-seq pipeline (v2.1.2; https://github.com/ENCODE-DCC/atac-seq-pipeline) was used for quality control and processing of ATAC-seq data using default parameters. The genomic TSVs version of this pipeline is v4. DARs were identified using DESeq2 (v1.42.1). Raw read counts per peak were modeled with a negative binomial distribution, and regions with an absolute value of log_2_ fold change > 2 and *p*-value < 0.001 were considered significantly differentially accessible.

#### RNA-seq library preparation and data analysis

VAHTS® Universal V8 RNA-seq Library Prep Kit for Illumina was used to prepare the RNA-seq library. Raw reads were assessed with FastQC (v0.11.9) using default settings. Quality filtering and adapter trimming were performed using fastp (v0.20.0) with parameters: --detect_adapter_for_pe, --cut_front, --cut_right, --trim_front1 = 10, and --trim_front2 = 10; other settings were default. Clean reads were aligned to the reference genome using HISAT2 (v2.2.1) with the --dta option. Gene-level quantification was conducted with featureCounts (v2.0.1) using -f -t gene -g gene_id; all other parameters were default. DEGs were identified using edgeR (v4.0.16), which models count data using the negative binomial distribution and estimates dispersion to account for biological variability. Genes with a absolute value of log_2_ fold change >0.58 and adjusted *p*-value <0.05 were considered significantly differentially expressed.

#### Definition and detection of srDMCs, srDARs, and srDEGs

LUAD is classified into three progressive stages: AIS, MIA, and IAC. To investigate dynamic molecular changes during tumor progression, we first calculated the alterations in specific attributes—namely DMCs, DARs, and DEGs—at each stage relative to the CTL group. Each attribute was then assigned to a stage based on the earliest time point at which a statistically significant change was detected. Based on the stage in which the change first occurred, attributes were categorized into six distinct groups. Once an attribute exhibited a significant change at a specific stage, its status in subsequent stages was no longer considered in comparison to CTL. As a result, the six categories were mutually exclusive, with no overlapping sites across them. For example, in the case of srDMCs, if a DMC first shows hypermethylation during the MIA stage, it is classified as “hyper in MIA” (Document S1).

#### Cell culture

The LUAD cell line A549 was acquired from the Cell Bank of the Chinese Academy of Medical Sciences (Shanghai, China). The cells were propagated in Dulbecco’s modified Eagle’s medium (DMEM, Gibco, Thermo Fisher Scientific) supplemented with 10% foetal bovine serum (FBS, Sigma-Aldrich) and 1% penicillin-streptomycin solution (Beyotime). A549 cells were cultured in a controlled environment incubator maintained at 37°C with 5% CO_2_.

#### Cell proliferation and colony formation assays

For the Cell Counting Kit-8 (CCK-8) assay, A549 cells treated with the c-Fos/AP-1 inhibitor T-5224 (MedChemExpress, Shanghai) were seeded in 96-well plates. Subsequently, CCK-8 reagent (Dojindo, Kumamoto, Japan) was added to achieve a final concentration of 10%, and the cells were incubated at 37°C for 3 h before measuring absorbance at a wavelength of 450 nm. These procedures were performed in triplicate to ensure experimental validity.

After treatment with T-5224, viable A549 cells were collected by trypsinization, washed with PBS, and resuspended in fresh complete medium. Cell viability was confirmed by trypan blue exclusion (>95% viability in all groups). The cells were then seeded at a density of 500 cells per well in 12-well plates and incubated at 37°C for one week. After incubation, the colonies were fixed with 4% paraformaldehyde for 10 min, stained with 0.1% crystal violet for 10 min, and counted. All experiments were performed in triplicate.

#### Transwell migration assay

Transwell migration assays were conducted as previously described.^68^ After treatment with T-5224, viable cells were collected by gentle trypsinization, washed twice with PBS, and resuspended in serum-free DMEM. Cell viability was confirmed to be >95% by trypan blue exclusion in all experimental groups.

An equal number of viable cells (5 × 104 cells/well) were placed in the upper chamber of the transwell insert in serum-free DMEM, while the lower chamber was filled with DMEM containing 10% FBS. The cells were incubated at 37°C for 24 h to facilitate cell migration. The migratory cells that reached the lower chamber were fixed with 4% paraformaldehyde for 10 min and stained with 0.1% crystal violet for 10 min before quantification.

#### Flow cytometry

Apoptosis was detected using an Annexin V-FITC/7-AAD Apoptosis Kit (Yeasen Biotechnology, Shanghai, China). A549 cells were collected after treatment with T-5224 for 24 h, centrifuged at 300 ×*g* for 5 min, washed, and resuspended in PBS. Annexin V-FITC and 7-AAD were added to the cell suspension according to the manufacturer’s instructions. Fluorescent samples of 10,000 cells were analysed by flow cytometry (BD Fortessa), and the data were analysed using FlowJo 10.0 software. All experiments were performed in triplicate.

#### Genome reference

The genome reference hg38 was used for processing WGBS, ATAC-seq, and RNA-seq data.

### Quantification and statistical analysis

All statistical analyses were performed using R4.3.2 statistical tests. Data are presented as mean ± standard deviation (SD), unless otherwise stated. The exact statistical test used (*n* values), statistical analysis methods (unpaired/paired two-tailed Student’s t-tests, Kruskal-Wallis H tests, and two-way Chi-squared test), and significance levels (ns: not significant, ∗*p* < 0.05, ∗∗*p* < 0.01, ∗∗∗*p* < 0.001, ∗∗∗∗*p* < 0.0001) are reported in the figures, figure legends, and tables.

## Supplemental information

Document S1. Figures S1–S4 and Tables S2, S4, S6, S8, S9, S11, and S13

Table S1. Summary of clinical information of subjects, related to Figure 1A

Table S3. Summary of WGBS data, related to Figure 1B

Table S5. DMCs identified in LUAD samples, related to Figure 2A (A) DMCs in the AIS vs. the CTL group. (B) DMCs in MIA vs. CTL group. (C) DMCs in IAC vs. CTL group. (D) Statistics of hyper and hypo DMCs in each group.

Table S7. srDMCs identified in LUAD samples, related to Figure 2C (A) srDMCs in AIS vs. CTL group. (B) srDMCs in MIA vs. CTL group. (C) srDMCs in IAC vs. CTL group. (D) Statistics of hyper and hypo srDMCs in each group.

Table S10. srDARs identified in LUAD samples, related to Figure 3C (A) srDARs in AIS vs. CTL group. (B) srDARs in MIA vs. CTL group. (C) srDARs in IAC vs. CTL group.

Table S12. Differentially expressed genes of LUAD samples, related to Figure 1A

## References

[1] Siegel R.L., Miller K.D., Fuchs H.E., Jemal A. (2022). Cancer statistics. CA Cancer J. Clin..

[2] Succony L., Rassl D.M., Barker A.P., McCaughan F.M., Rintoul R.C. (2021). Adenocarcinoma spectrum lesions of the lung: Detection, pathology and treatment strategies. Cancer Treat Rev..

[3] Lambe G., Durand M., Buckley A., Nicholson S., McDermott R. (2020). Adenocarcinoma of the lung: from BAC to the future. Insights Imaging.

[4] Hu X., Estecio M.R., Chen R., Reuben A., Wang L., Fujimoto J., Carrot-Zhang J., McGranahan N., Ying L., Fukuoka J. (2021). Evolution of DNA methylome from precancerous lesions to invasive lung adenocarcinomas. Nat. Commun..

[5] Karasaki T., Moore D.A., Veeriah S., Naceur-Lombardelli C., Toncheva A., Magno N., Ward S., Bakir M.A., Watkins T.B.K., Grigoriadis K. (2023). Evolutionary characterization of lung adenocarcinoma morphology in TRACERx. Nat. Med..

[6] Hu X., Fujimoto J., Ying L., Fukuoka J., Ashizawa K., Sun W., Reuben A., Chow C.W., McGranahan N., Chen R. (2019). Multi-region exome sequencing reveals genomic evolution from preneoplasia to lung adenocarcinoma. Nat. Commun..

[7] Tremblay M.W., Jiang Y.H. (2019). DNA Methylation and Susceptibility to Autism Spectrum Disorder. Annu. Rev. Med..

[8] Xi Y., Lin Y., Guo W., Wang X., Zhao H., Miao C., Liu W., Liu Y., Liu T., Luo Y. (2022). Multi-omic characterization of genome-wide abnormal DNA methylation reveals diagnostic and prognostic markers for esophageal squamous-cell carcinoma. Signal Transduct. Target. Ther..

[9] Portela A., Esteller M. (2010). Epigenetic modifications and human disease. Nat. Biotechnol..

[10] Noro R., Ishigame T., Walsh N., Shiraishi K., Robles A.I., Ryan B.M., Schetter A.J., Bowman E.D., Welsh J.A., Seike M. (2017). A Two-Gene Prognostic Classifier for Early-Stage Lung Squamous Cell Carcinoma in Multiple Large-Scale and Geographically Diverse Cohorts. J. Thorac. Oncol..

[11] Zhao L., Wu X., Zheng J., Dong D. (2021). DNA methylome profiling of circulating tumor cells in lung cancer at single base-pair resolution. Oncogene.

[12] Yu H., Raut J.R., Schöttker B., Holleczek B., Zhang Y., Brenner H. (2020). Individual and joint contributions of genetic and methylation risk scores for enhancing lung cancer risk stratification: data from a population-based cohort in Germany. Clin. Epigenetics.

[13] Zhao S.G., Chen W.S., Li H., Foye A., Zhang M., Sjöström M., Aggarwal R., Playdle D., Liao A., Alumkal J.J. (2020). The DNA methylation landscape of advanced prostate cancer. Nat. Genet..

[14] Klughammer J., Kiesel B., Roetzer T., Fortelny N., Nemc A., Nenning K.H., Furtner J., Sheffield N.C., Datlinger P., Peter N. (2018). The DNA methylation landscape of glioblastoma disease progression shows extensive heterogeneity in time and space. Nat. Med..

[15] Duruisseaux M., Martínez-Cardús A., Calleja-Cervantes M.E., Moran S., Castro de Moura M., Davalos V., Piñeyro D., Sanchez-Cespedes M., Girard N., Brevet M. (2018). Epigenetic prediction of response to anti-PD-1 treatment in non-small-cell lung cancer: a multicentre, retrospective analysis. Lancet Respir. Med..

[16] Jurmeister P., Bockmayr M., Seegerer P., Bockmayr T., Treue D., Montavon G., Vollbrecht C., Arnold A., Teichmann D., Bressem K. (2019). Machine learning analysis of DNA methylation profiles distinguishes primary lung squamous cell carcinomas from head and neck metastases. Sci. Transl. Med..

[17] Vaissiere T., Hung R.J., Zaridze D., Moukeria A., Cuenin C., Fasolo V., Ferro G., Paliwal A., Hainaut P., Brennan P., Tost J. (2008). Quantitative Analysis of DNA Methylation Profiles in Lung Cancer Identifies Aberrant DNA Methylation of Specific Genes and Its Association with Gender and Cancer Risk Factors. Cancer Res..

[18] Klemm S.L., Shipony Z., Greenleaf W.J. (2019). Chromatin accessibility and the regulatory epigenome. Nat. Rev. Genet..

[19] Corces M.R., Granja J.M., Shams S., Louie B.H., Seoane J.A., Zhou W., Silva T.C., Groeneveld C., Wong C.K., Cho S.W. (2018). The chromatin accessibility landscape of primary human cancers. Science.

[20] Xin J., Zhang H., He Y., Duren Z., Bai C., Chen L., Luo X., Yan D.S., Zhang C., Zhu X. (2020). Chromatin accessibility landscape and regulatory network of high-altitude hypoxia adaptation. Nat. Commun..

[21] Thurman R.E., Rynes E., Humbert R., Vierstra J., Maurano M.T., Haugen E., Sheffield N.C., Stergachis A.B., Wang H., Vernot B. (2012). The accessible chromatin landscape of the human genome. Nature.

[22] Wang Z., Tu K., Xia L., Luo K., Luo W., Tang J., Lu K., Hu X., He Y., Qiao W. (2019). The Open Chromatin Landscape of Non–Small Cell Lung Carcinoma. Cancer Res..

[23] Nouruzi S., Ganguli D., Tabrizian N., Kobelev M., Sivak O., Namekawa T., Thaper D., Baca S.C., Freedman M.L., Aguda A. (2022). ASCL1 activates neuronal stem cell-like lineage programming through remodeling of the chromatin landscape in prostate cancer. Nat. Commun..

[24] Kusi M., Zand M., Lin L.L., Chen M., Lopez A., Lin C.L., Wang C.M., Lucio N.D., Kirma N.B., Ruan J. (2022). 2-Hydroxyglutarate destabilizes chromatin regulatory landscape and lineage fidelity to promote cellular heterogeneity. Cell Rep..

[25] Lister R., Pelizzola M., Dowen R.H., Hawkins R.D., Hon G., Tonti-Filippini J., Nery J.R., Lee L., Ye Z., Ngo Q.M. (2009). Human DNA methylomes at base resolution show widespread epigenomic differences. Nature.

[26] Berman B.P., Weisenberger D.J., Aman J.F., Hinoue T., Ramjan Z., Liu Y., Noushmehr H., Lange C.P.E., van Dijk C.M., Tollenaar R.A.E.M. (2011). Regions of focal DNA hypermethylation and long-range hypomethylation in colorectal cancer coincide with nuclear lamina-associated domains. Nat. Genet..

[27] Buenrostro J.D., Giresi P.G., Zaba L.C., Chang H.Y., Greenleaf W.J. (2013). Transposition of native chromatin for fast and sensitive epigenomic profiling of open chromatin, DNA-binding proteins and nucleosome position. Nat. Methods.

[28] Ronkina N., Gaestel M. (2022). MAPK-Activated Protein Kinases: Servant or Partner?. Annu. Rev. Biochem..

[29] Braicu C., Buse M., Busuioc C., Drula R., Gulei D., Raduly L., Rusu A., Irimie A., Atanasov A.G., Slaby O. (2019). A Comprehensive Review on MAPK: A Promising Therapeutic Target in Cancer. Cancers.

[30] Molina J.R., Adjei A.A. (2006). The Ras/Raf/MAPK Pathway. J. Thorac. Oncol..

[31] Lee J.T., McCubrey J.A. (2002). The Raf/MEK/ERK signal transduction cascade as a target for chemotherapeutic intervention in leukemia. Leukemia.

[32] Nakano Y., Shimizu W. (2022). Brugada Syndrome as a Major Cause of Sudden Cardiac Death in Asians. JACC. Asia.

[33] Dong X., Guo R., Ji T., Zhang J., Xu J., Li Y., Sheng Y., Wang Y., Fang K., Wen Y. (2022). YY1 safeguard multidimensional epigenetic landscape associated with extended pluripotency. Nucleic Acids Res..

[34] Fang S., Li J., Xiao Y., Lee M., Guo L., Han W., Li T., Hill M.C., Hong T., Mo W. (2019). Tet inactivation disrupts YY1 binding and long-range chromatin interactions during embryonic heart development. Nat. Commun..

[35] Xu C., Corces V.G. (2018). Nascent DNA methylome mapping reveals inheritance of hemimethylation at CTCF/cohesin sites. Science.

[36] Katz M., Amit I., Yarden Y. (2007). Regulation of MAPKs by growth factors and receptor tyrosine kinases. Biochim. Biophys. Acta.

[37] Sharma S.V., Bell D.W., Settleman J., Haber D.A. (2007). Epidermal growth factor receptor mutations in lung cancer. Nat. Rev. Cancer.

[38] Qi M., Fan S., Huang M., Pan J., Li Y., Miao Q., Lyu W., Li X., Deng L., Qiu S. (2022). Targeting FAPα-expressing hepatic stellate cells overcomes resistance to antiangiogenics in colorectal cancer liver metastasis models. J. Clin. Investig..

[39] McLean C.Y., Bristor D., Hiller M., Clarke S.L., Schaar B.T., Lowe C.B., Wenger A.M., Bejerano G. (2010). GREAT improves functional interpretation of cis-regulatory regions. Nat. Biotechnol..

[40] Tanigawa Y., Dyer E.S., Bejerano G. (2022). WhichTF is functionally important in your open chromatin data?. PLoS Comput. Biol..

[41] Whitsett J.A., Wert S.E., Weaver T.E. (2010). Alveolar Surfactant Homeostasis and the Pathogenesis of Pulmonary Disease. Annu. Rev. Med..

[42] Heinz S., Benner C., Spann N., Bertolino E., Lin Y.C., Laslo P., Cheng J.X., Murre C., Singh H., Glass C.K. (2010). Simple combinations of lineage-determining transcription factors prime cis-regulatory elements required for macrophage and B cell identities. Mol. Cell.

[43] Eferl R., Wagner E.F. (2003). AP-1: a double-edged sword in tumorigenesis. Nat. Rev. Cancer.

[44] Bakiri L., Hasenfuss S.C., Guío-Carrión A., Thomsen M.K., Hasselblatt P., Wagner E.F. (2024). Liver cancer development driven by the AP-1/c-Jun∼Fra-2 dimer through c-Myc. Proc. Natl. Acad. Sci. USA.

[45] Kyriakis J.M. (1999). Activation of the AP-1 Transcription Factor by Inflammatory Cytokines of the TNF Family. Gene Expr..

[46] Ozolins T.R., Hales B.F. (1999). Post-translational regulation of AP-1 transcription factor DNA-binding activity in the rat conceptus. Mol. Pharmacol..

[47] Albertina, B. TCGA-LUAD. The Cancer Imaging Archive (TCIA) https://www.cancerimagingarchive.net/collection/tcga-luad/(2016).

[48] Lan C.-N., Cai W.-J., Shi J., Yi Z.-J. (2021). MAPK inhibitors protect against early-stage osteoarthritis by activating autophagy. Mol. Med. Rep..

[49] Ou S., Chen H., Wang H., Ye J., Liu H., Tao Y., Ran S., Mu X., Liu F., Zhu S. (2023). Fusobacterium nucleatum upregulates MMP7 to promote metastasis-related characteristics of colorectal cancer cell via activating MAPK(JNK)-AP1 axis. J. Transl. Med..

[50] Su Z., Yang Z., Xu Y., Chen Y., Yu Q. (2015). Apoptosis, autophagy, necroptosis, and cancer metastasis. Mol. Cancer.

[51] Tang Z., Kang B., Li C., Chen T., Zhang Z. (2019). GEPIA2: an enhanced web server for large-scale expression profiling and interactive analysis. Nucleic Acids Res..

[52] Lee H.-J., Lee C.H., Jeong Y.J., Chung D.H., Goo J.M., Park C.M., Austin J.H.M. (2012). IASLC/ATS/ERS International Multidisciplinary Classification of Lung Adenocarcinoma: novel concepts and radiologic implications. J. Thorac. Imaging.

[53] Zhu J., Wang W., Xiong Y., Xu S., Chen J., Wen M., Zhao Y., Lei J., Jiang T. (2023). Evolution of lung adenocarcinoma from preneoplasia to invasive adenocarcinoma. Cancer Med..

[54] Wang S., Meng F., Li M., Bao H., Chen X., Zhu M., Liu R., Xu X., Yang S., Wu X. (2023). Multidimensional Cell-Free DNA Fragmentomic Assay for Detection of Early-Stage Lung Cancer. Am. J. Respir. Crit. Care Med..

[55] Stadler M.B., Murr R., Burger L., Ivanek R., Lienert F., Schöler A., van Nimwegen E., Wirbelauer C., Oakeley E.J., Gaidatzis D. (2011). DNA-binding factors shape the mouse methylome at distal regulatory regions. Nature.

[56] Kaluscha S., Domcke S., Wirbelauer C., Stadler M.B., Durdu S., Burger L., Schübeler D. (2022). Evidence that direct inhibition of transcription factor binding is the prevailing mode of gene and repeat repression by DNA methylation. Nat. Genet..

[57] Carter B., Zhao K. (2021). The epigenetic basis of cellular heterogeneity. Nat. Rev. Genet..

[58] Kim J., Minna J.D. (2022). AP-1 leads the way in lung cancer transformation. Dev. Cell.

[59] Kadur Lakshminarasimha Murthy P., Xi R., Arguijo D., Everitt J.I., Kocak D.D., Kobayashi Y., Bozec A., Vicent S., Ding S., Crawford G.E. (2022). Epigenetic basis of oncogenic-Kras-mediated epithelial-cellular proliferation and plasticity. Dev. Cell.

[60] Papavassiliou A.G., Musti A.M. (2020). The Multifaceted Output of c-Jun Biological Activity: Focus at the Junction of CD8 T Cell Activation and Exhaustion. Cells.

[61] Du K., Sun S., Jiang T., Liu T., Zuo X., Xia X., Liu X., Wang Y., Bu Y. (2022). E2F2 promotes lung adenocarcinoma progression through B-Myb- and FOXM1-facilitated core transcription regulatory circuitry. Int. J. Biol. Sci..

[62] Cheung W.K.C., Zhao M., Liu Z., Stevens L.E., Cao P.D., Fang J.E., Westbrook T.F., Nguyen D.X. (2013). Control of alveolar differentiation by the lineage transcription factors GATA6 and HOPX inhibits lung adenocarcinoma metastasis. Cancer Cell.

[63] Liu W.J., Du Y., Wen R., Yang M., Xu J. (2020). Drug resistance to targeted therapeutic strategies in non-small cell lung cancer. Pharmacol. Ther..

[64] Roberts P.J., Der C.J. (2007). Targeting the Raf-MEK-ERK mitogen-activated protein kinase cascade for the treatment of cancer. Oncogene.

[65] Sun D., Xi Y., Rodriguez B., Park H.J., Tong P., Meong M., Goodell M.A., Li W. (2014). MOABS: model based analysis of bisulfite sequencing data. Genome Biol..

[66] Chen S., Zhou Y., Chen Y., Gu J. (2018). fastp: an ultra-fast all-in-one FASTQ preprocessor. Bioinformatics.

[67] Kim D., Paggi J.M., Park C., Bennett C., Salzberg S.L. (2019). Graph-based genome alignment and genotyping with HISAT2 and HISAT-genotype. Nat. Biotechnol..

[68] Kota P., Terrell E.M., Ritt D.A., Insinna C., Westlake C.J., Morrison D.K. (2019). M-Ras/Shoc2 signaling modulates E-cadherin turnover and cell–cell adhesion during collective cell migration. Proc. Natl. Acad. Sci. USA.

