# Supplementary material for: Epigenetic-mediated positive feedback loop facilitates the progression of lung adenocarcinoma
Source: iScience. 2025 Aug 26;28(10):113339. doi: 10.1016/j.isci.2025.113339 (PMC12475578; doi:10.1016/j.isci.2025.113339)
Supplement: Document S1. Figures S1–S4 and Tables S2, S4, S6, S8, S9, S11, and S13 [file mmc1.pdf]

iScience, Volume *28*

Supplemental information

Epigenetic-mediated positive feedback loop facilitates the progression of lung adenocarcinoma

Yihang Cheng, Yifeng Luo, Tingting Hu, Qiaoling Ren, Li Xu, Tuo Yi, Yuan Tan, Wei Li, Xuxin Wang, Yaoxiang Sun, Mingzhi Chen, Zhonghua Shen, Bin Zhang, Youhuang Bai, Yue Tao, Zhihong Cao, and Deqiang Sun

Supplemental figures

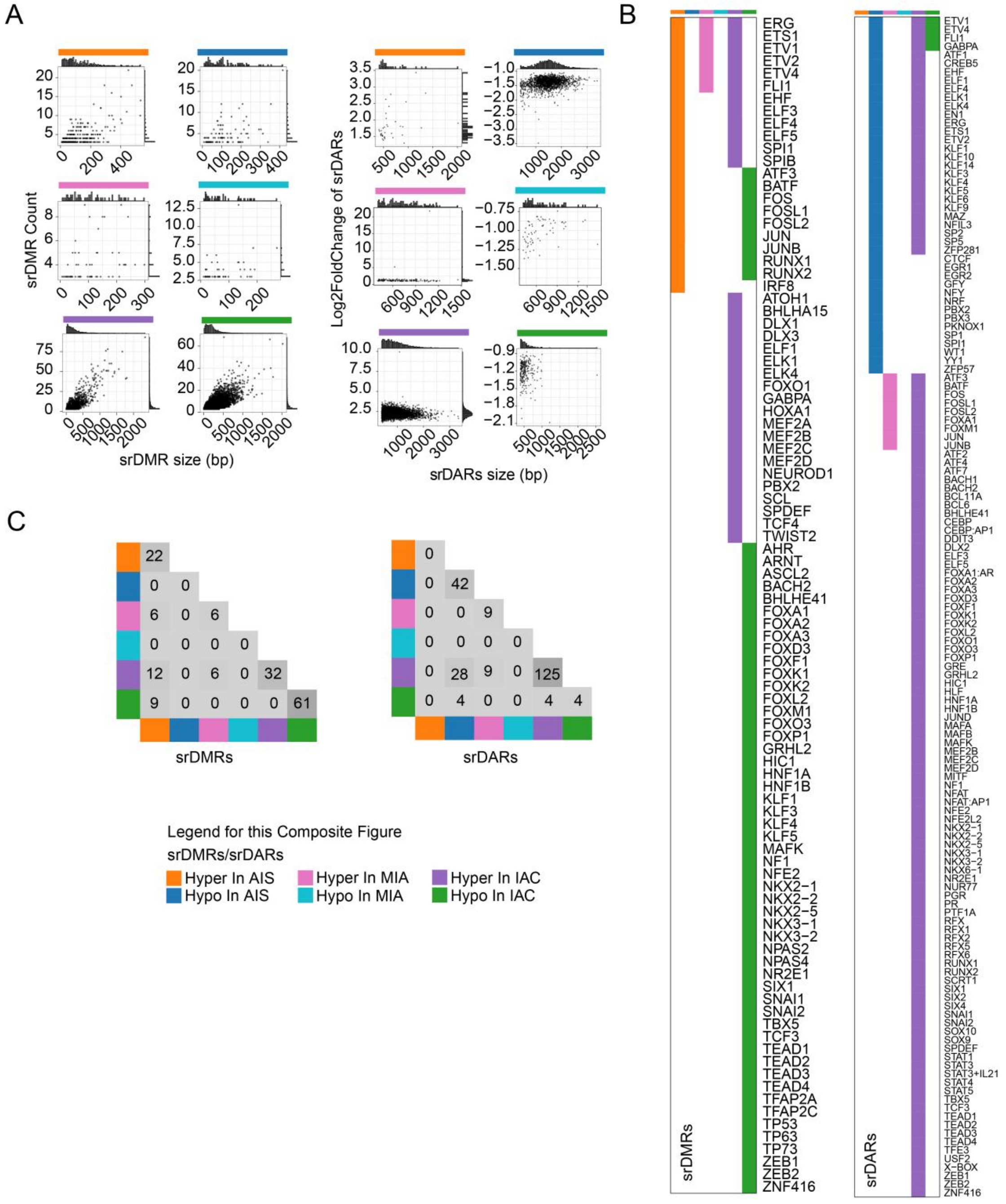

Figure S1. Transcription factors enriched in srDMRs and srDARs

A. Left: Distribution of srDMR size and count across AIS, MIA, and IAC stages. Right: Distribution of srDAR size and changes in chromatin accessibility across AIS, MIA, and IAC stages. **B.** Identification of TFs enriched within srDMRs (left) and srDARs (right) at different stages of LUAD, as determined by HOMER analysis. **C.** Number of TFs identified at each stage of disease progression.

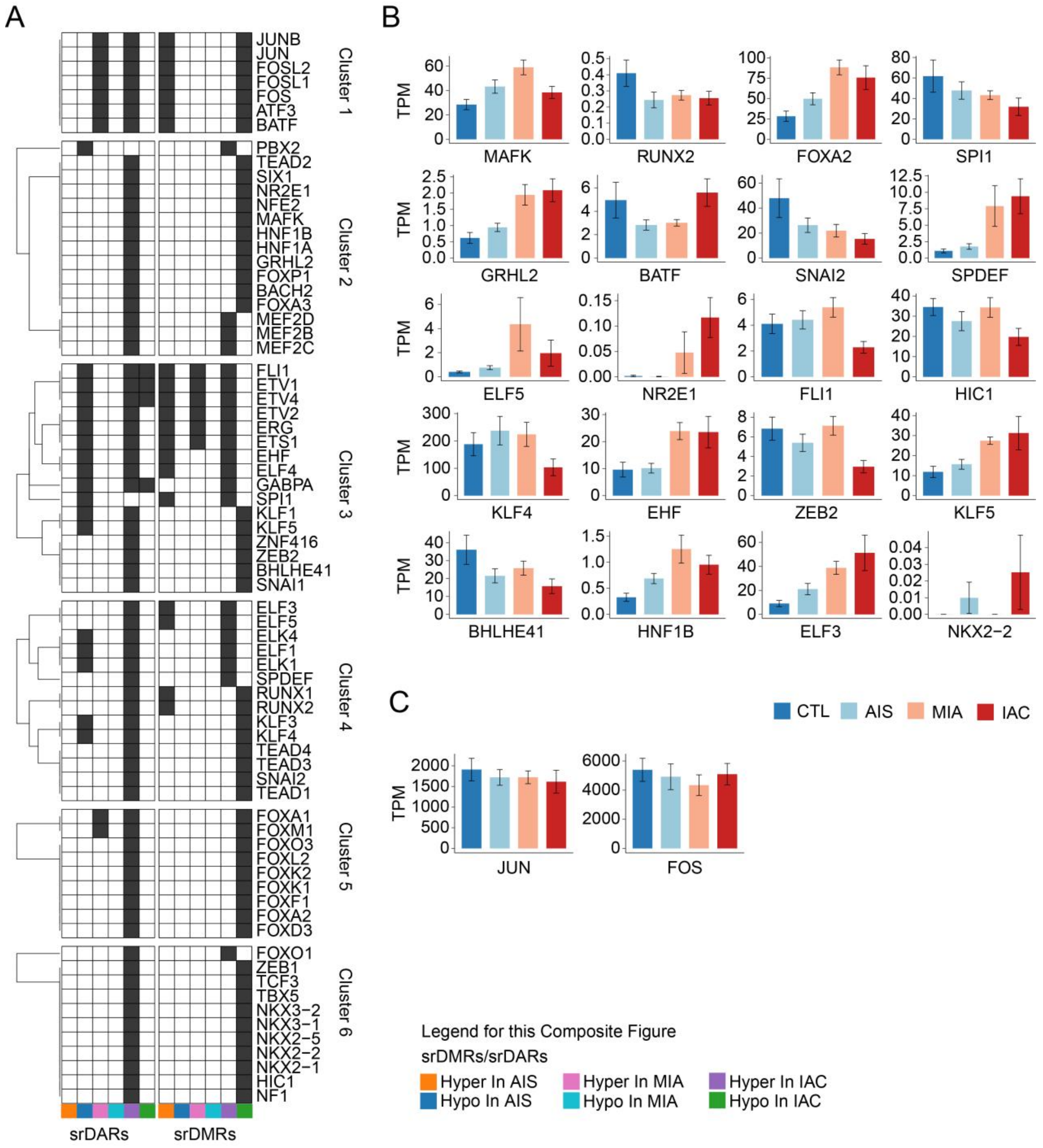

Figure S2. Epi TFs in LUAD

A. Classification of epi TFs in LUAD. **B.** Expression levels (mean ± SD) of differentially expressed epi TFs across LUAD stages. **C.** Expression levels (mean ± SD) of JUN and FOS.

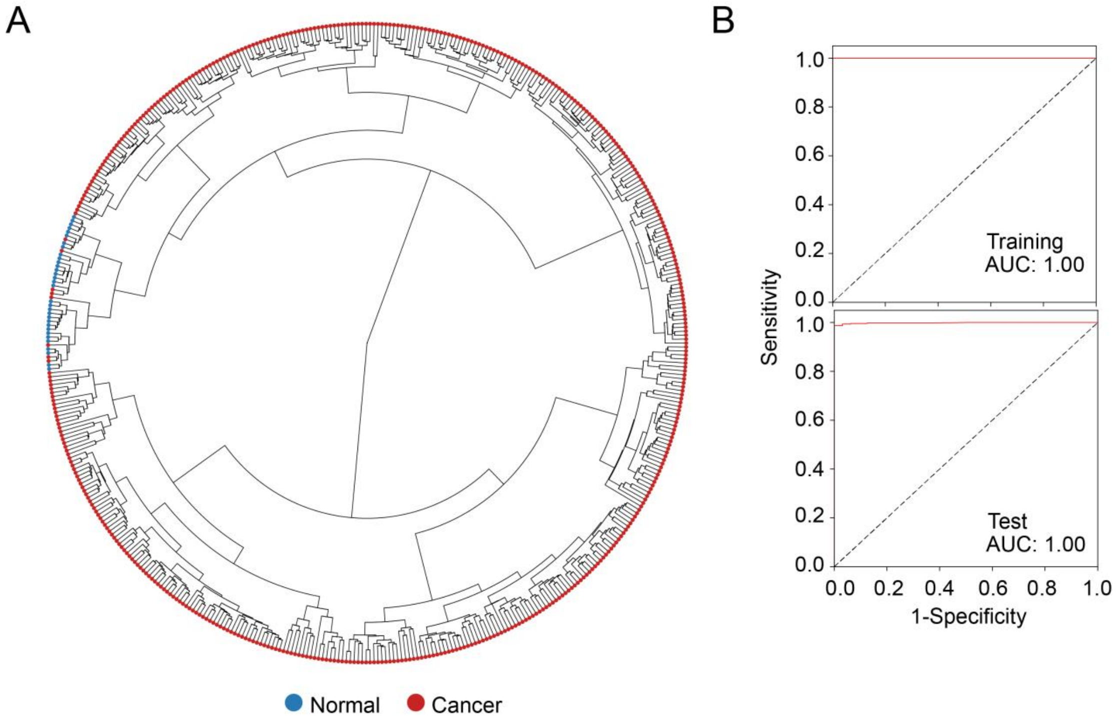

Figure S3. Methylation-Based Classification Models in TCGA-LUAD Analysis

A. Utilisation of methylation profiles from 1,416 shared srDARs and srDMRs for hierarchical clustering of TCGA-LUAD samples. **B.** ROC curves for a binary classification model adept at distinguishing between cancerous and normal tissues within the TCGA-LUAD datasets, with separate curves for training (top) and test (bottom) sets.

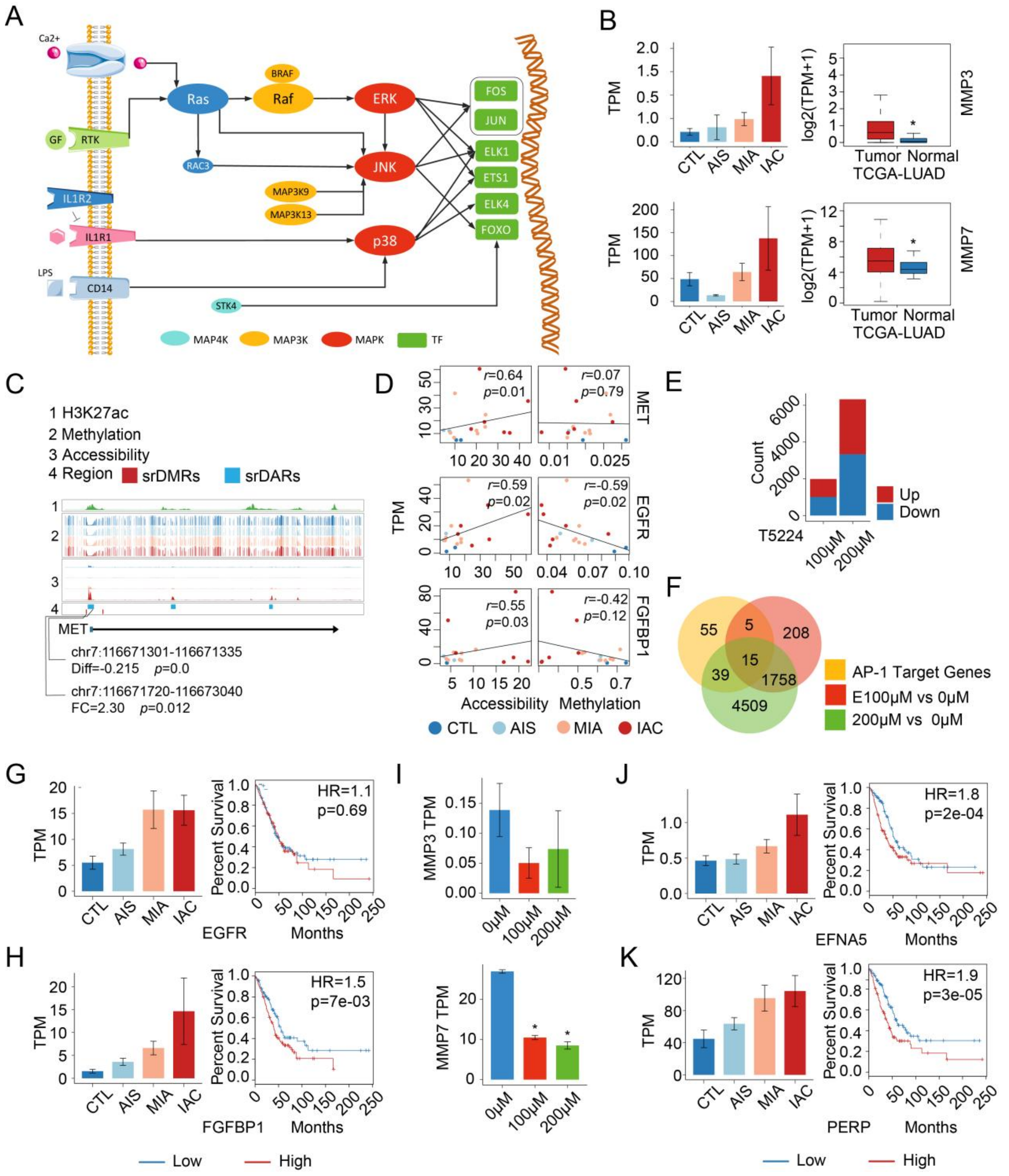

Figure S4. Downstream genes directly regulated by AP-1

A. A streamlined overview of the MAPK signalling pathway and its downstream epi TFs in LUAD. **B.** Expression levels of MMP3 (top) and MMP7 (bottom) in each stage of LUAD (left) and TCGA-LUAD (right). **C.** Methylation levels and chromatin accessibility profiles near MET regulatory regions. Green peaks indicate regions of active transcription at promoters or enhancers. **D.** The correlation between gene expression and chromatin accessibility (and methylation level) at AP-1 binding sites in promoter regions of MET, EGFR and FGFBP1. **E.** DEG numbers of A549 cells treated with different concentrations of T-5224 (left: 0 μM vs 100 μM, right: 0 μM vs 200 μM)**.** **F.** Venn diagram of different gene sets. AP-1: DEGs regulated by AP-1 in lung cancer tissue; 100 μM vs 0 μM: DEGs of A549 cells treated with 0 μM and 100 μM T-5224; 200 μM vs 0 μM: DEGs of A549 cells treated with 0 μM and 200 μM T-5224. **G, H.**

Expression levels (mean ± SD) and survival curves (from GEPIA2) of EGFR (G) and FGFBP1 (H) in LUAD. **I.** Expression levels (mean ± SD, n=3) of MMP3 (top) and MMP7 (bottom) of A549 cells across a range of T-5224 concentrations (0 μM, 100 μM, and 200 μM). Statistical comparisons were made using unpaired two-tailed Student's t-test (* $p < 0.05$, ** $p < 0.01$, *** $p < 0.001$, **** $p < 0.0001$). **J, K.** Expression levels (mean ± SD) and survival curves (from GEPIA2) of EFNA5 (J) and PERP (K) in LUAD.

Supplemental tables

Table S2. Summary of clinical characteristics associated with different subtypes of LUAD

	Age (median/IQR)	Smoking (No/yes)	Sex (female/male)	
			all	cancer
**CTL**	57 / 16.2	18 / 7	12 / 20	-
**AIS**	60 / 17.5	30 / 5	28 / 10	28 / 10
**MIA**	63 / 13	28 / 7	22 / 16	22 / 15
**IAC**	64.5 / 15.8	31 / 3	26 / 18	16 / 18
**statistical tests**	Kruskal-Wallis H test	two-way Chi-squared test	two-way Chi-squared test	
**p-value**	0.1879 (ns)	0.0759 (ns)	0.02415 (*)	0.3114 (ns)

Table S4. The Average Methylation Level of Specific Genomic Regions, related to Figure 1D

GenomicRange	Group	Mean	SD	CV	IQR
**CpG Islands**	CTL	0.298	0.008	0.026	0.011
**CpG Islands**	AIS	0.299	0.007	0.025	0.006
**CpG Islands**	MIA	0.300	0.009	0.030	0.011
**CpG Islands**	IAC	0.310	0.016	0.052	0.018
**CpG Sea**	CTL	0.824	0.012	0.014	0.017
**CpG Sea**	AIS	0.834	0.006	0.008	0.009
**CpG Sea**	MIA	0.831	0.008	0.010	0.013
**CpG Sea**	IAC	0.803	0.040	0.049	0.032
**CpG Shelves**	CTL	0.808	0.012	0.014	0.016
**CpG Shelves**	AIS	0.817	0.006	0.008	0.008
**CpG Shelves**	MIA	0.814	0.008	0.010	0.013
**CpG Shelves**	IAC	0.790	0.037	0.046	0.029
**CpG Shores**	CTL	0.606	0.009	0.014	0.010
**CpG Shores**	AIS	0.610	0.008	0.013	0.008
**CpG Shores**	MIA	0.607	0.007	0.012	0.010
**CpG Shores**	IAC	0.596	0.030	0.050	0.022
**Exons**	CTL	0.719	0.010	0.014	0.015
**Exons**	AIS	0.728	0.007	0.009	0.006
**Exons**	MIA	0.726	0.007	0.009	0.009
**Exons**	IAC	0.707	0.034	0.048	0.029
**Intergenic**	CTL	0.671	0.012	0.018	0.019
**Intergenic**	AIS	0.681	0.009	0.013	0.008
**Intergenic**	MIA	0.677	0.009	0.013	0.011
**Intergenic**	IAC	0.646	0.041	0.063	0.037
**Intron**	CTL	0.781	0.011	0.015	0.018
**Intron**	AIS	0.792	0.006	0.008	0.007
**Intron**	MIA	0.789	0.007	0.009	0.011
**Intron**	IAC	0.767	0.034	0.045	0.029
**Promoter 1K**	CTL	0.651	0.009	0.014	0.014
**Promoter 1K**	AIS	0.659	0.007	0.010	0.007
**Promoter 1K**	MIA	0.657	0.007	0.010	0.008
**Promoter 1K**	IAC	0.638	0.031	0.048	0.024
**Promoter 5k**	CTL	0.758	0.010	0.014	0.015
**Promoter 5k**	AIS	0.768	0.007	0.009	0.007
**Promoter 5k**	MIA	0.765	0.007	0.010	0.010
**Promoter 5k**	IAC	0.746	0.034	0.046	0.027
**TSS**	CTL	0.200	0.008	0.042	0.007
**TSS**	AIS	0.206	0.019	0.092	0.007

**TSS**	MIA	0.201	0.008	0.038	0.006
**TSS**	IAC	0.202	0.011	0.055	0.015
**3'UTR**	CTL	0.834	0.009	0.011	0.010
**3'UTR**	AIS	0.839	0.005	0.006	0.006
**3'UTR**	MIA	0.839	0.006	0.007	0.008
**3'UTR**	IAC	0.826	0.027	0.033	0.019
**5'UTR**	CTL	0.376	0.007	0.019	0.007
**5'UTR**	AIS	0.382	0.014	0.035	0.007
**5'UTR**	MIA	0.378	0.007	0.018	0.008
**5'UTR**	IAC	0.374	0.018	0.047	0.012

Table S6. DMCs in different genomic regions of different group in LUAD

Region	P-value	group	DMCs				Total
			Hyper		Hypo		
			Observation	Residuals	Observation	Residuals	
**cgIslands**	<2.2e-16 (****)	**CTL.vs.AIS**	**398**	**-8.7**	**392**	**16.2**	**790**
		**CTL.vs.MIA**	**223**	**-6.8**	**229**	**12.8**	**452**
		**CTL.vs.IAC**	**7760**	**4.0**	**1786**	**-7.5**	**9546**
**cgSea**	<2.2e-16 (****)	CTL.vs.AIS	5772	169.1	2155	-58.5	7927
		CTL.vs.MIA	4638	140.4	2487	-48.6	7125
		CTL.vs.IAC	8023	-67.8	149278	23.5	157301
**cgShelves**	<2.2e-16 (****)	CTL.vs.AIS	365	25.9	180	-12.5	545
		CTL.vs.MIA	265	20.1	173	-9.7	438
		CTL.vs.IAC	971	-11.8	6560	5.7	7531
**cgShores**	<2.2e-16 (****)	CTL.vs.AIS	665	22.5	305	-14.5	970
		CTL.vs.MIA	464	14.9	337	-9.6	801
		CTL.vs.IAC	3841	-9.1	11316	5.9	15157
**exons**	<2.2e-16 (****)	CTL.vs.AIS	247	11.4	65	-9.1	312
		CTL.vs.MIA	193	7.3	101	-5.9	294
		CTL.vs.IAC	2345	-4.0	4205	3.2	6550
**intergenic**	<2.2e-16 (****)	CTL.vs.AIS	3461	107.0	1666	-41.7	5127
		CTL.vs.MIA	2968	94.4	1720	-36.8	4688
		CTL.vs.IAC	6986	-46.6	84786	18.2	91772
**intron**	<2.2e-16 (****)	CTL.vs.AIS	2955	84.1	1132	-38.6	4087
		CTL.vs.MIA	2165	61.9	1402	-28.4	3567
		CTL.vs.IAC	11374	-30.8	75679	14.1	87053
**promoter.1k**	<2.2e-16 (****)	CTL.vs.AIS	733	14.9	340	-12.1	1073
		CTL.vs.MIA	468	5.9	433	-4.8	901
		CTL.vs.IAC	8163	-4.5	13505	3.7	21668
**promoter.5k**	<2.2e-16 (****)	CTL.vs.AIS	1190	24.3	588	-17.3	1778
		CTL.vs.MIA	665	10.2	674	-7.2	1339
		CTL.vs.IAC	9711	-7.9	21686	5.6	31397
**utr3**	<2.2e-16 (****)	CTL.vs.AIS	223	14.7	71	-9.5	294
		CTL.vs.MIA	141	9.4	81	-6.1	222
		CTL.vs.IAC	956	-6.2	3026	4.0	3982
**utr5**	<4.6e-12 (****)	CTL.vs.AIS	135	3.6	55	-3.7	190
		CTL.vs.MIA	80	3.0	30	-3.1	110
		CTL.vs.IAC	938	-1.8	966	1.9	1904

Note: Two-way Chi-squared test was used for statistical testing

Table S8. The srDMRs in LUAD samples, related to Figure 2D

srDMR		cgIslands	cgShores	cgShelves	cgSea	exons	intergenic	intron	promoter.1k	promoter.5k	utr3	utr5
HyperInAIS	1044	35	53	23	204	95	90	202	103	181	18	21
HypoInAIS	344	37	20	12	33	25	25	76	34	63	6	8
HyperInMIA	199	9	17	5	28	18	13	36	24	41	2	4
HypoInMIA	161	11	6	5	28	12	17	34	12	31	4	1
HyperInIAC	6189	641	447	96	605	603	530	1062	670	1120	106	148
HypoInIAC	36562	171	1278	686	11460	1486	5995	7904	1894	4828	369	205

Table S9. Summary of ATAC-seq data, related to Figure 1A

ID	SampleName	NumberOfPeaks	TotalReads	PercentageOfMappedReads	TotalNoDupReads	frac_reads_in_nfr	NRF	PBC1	PBC2	tss_enrich
64	LNM_64	298390	146258296	98.7%	95211938	0.5387	0.8191	0.8131	5.1325	6.0363
74	LNM_74	298518	156128610	99.1%	103060456	0.4007	0.8031	0.8007	4.8929	7.7638
78	LNM_78	298441	145243212	98.9%	92466800	0.3604	0.7969	0.7955	4.7768	7.6886
9	AIS_9	298078	130351108	98.9%	89414116	0.5743	0.8571	0.8536	6.6263	2.4775
32	AIS_32	298361	144385090	99.1%	96081012	0.5909	0.8255	0.8230	5.5036	4.3852
55	MIA_55	297786	199558512	98.9%	138424540	0.3435	0.8408	0.8364	5.9053	3.3043
76	MIA_76	265732	140500154	99.0%	87487018	0.3820	0.8011	0.8004	4.8856	7.2935
14	MIA_14	298457	146458550	98.9%	97308158	0.3826	0.8414	0.8410	6.1320	9.2277
13	MIA_13	297471	171711710	98.4%	115833134	0.4033	0.8444	0.8418	6.1350	3.6036
15	MIA_15	298682	149414082	99.2%	78667280	0.4440	0.8291	0.8418	6.1810	8.0384
34	MIA_34	294742	144616128	99.2%	89471294	0.4387	0.8151	0.8171	5.3567	8.9855
40	MIA_40	298247	142270784	99.1%	89626176	0.4795	0.8033	0.8010	4.8731	9.1151
56	IAC_56	298505	154333288	98.9%	99928528	0.3819	0.8100	0.8067	5.0145	12.3351
60	IAC_60	298150	128136082	98.8%	80707184	0.4386	0.8289	0.8269	5.6133	5.6251
63	IAC_63	298419	147588696	99.0%	95102840	0.4482	0.8244	0.8223	5.4561	6.2392
65	IAC_65	298192	197200160	98.8%	131409728	0.4169	0.8287	0.8271	5.6578	9.1193
70	IAC_70	260965	162354580	99.1%	85035278	0.4116	0.8175	0.8302	5.7772	14.7289
85	IAC_85	298501	165448902	99.1%	106542396	0.4481	0.7992	0.7961	4.7659	11.0985
23	IAC_23	236945	117161004	99.4%	56296208	0.3369	0.7888	0.8081	5.1495	17.7266
37	IAC_37	285171	155894836	99.2%	100854318	0.3732	0.7961	0.7949	4.7564	14.9331

Table S11. Summary of RNA-seq data, related to Figure 1A

ID	SampleName	MbOfBases(>Q30)	PercentageOFreads(>Q30)	PercentageOfReadsPassingFastp	MbOfReads	MbReadsOfMapped	AlignmentRate	PercentageOfAssignedReads	MbOfAssignedReads
41	CTL_41	6700.2	94.70%	98.40%	57.9	56.8	97.80%	66.30%	38.4
64	CTL_64	8157.2	94.90%	98.20%	76.3	71.7	92.80%	49.90%	38.1
74	CTL_74	8034.3	95.00%	98.40%	71.7	69.5	96.60%	60.60%	43.5
154	CTL_154	8101.6	95.20%	98.50%	74.6	73.2	97.80%	59.10%	44.1
174	CTL_174	8995.9	95.20%	98.60%	80.5	79.1	98.00%	62.50%	50.3
181	CTL_181	7986.4	95.40%	98.70%	71.6	70.2	97.70%	60.50%	43.3
183	CTL_183	9184.1	95.20%	98.50%	81.9	80.2	97.60%	62.50%	51.2
228	CTL_228	7721.6	95.30%	98.60%	67.9	66.5	97.80%	64.10%	43.5
290	CTL_290	8401	95.00%	98.20%	79	75.1	94.20%	53.60%	42.3
9	AIS_9	7131.8	94.70%	98.20%	62.3	61.2	98.00%	65.90%	41
32	AIS_32	7631.3	94.60%	98.20%	66.5	65.1	97.70%	66.00%	43.9
95	AIS_95	8500.6	95.70%	98.90%	73.3	72.1	98.20%	65.20%	47.8
103	AIS_103	10083.2	95.70%	98.90%	87.2	85.6	98.10%	65.10%	56.8
129	AIS_129	6566.3	93.70%	98.00%	58.4	57.4	98.10%	65.90%	38.5
136	AIS_136	8129.9	94.90%	98.40%	72.7	71.2	97.70%	65.40%	47.5
138	AIS_138	9279.4	95.00%	98.40%	84.5	82.9	97.90%	61.30%	51.8
145	AIS_145	8184.4	95.10%	98.40%	73.1	71.7	97.80%	62.60%	45.7
189	AIS_189	7470	93.80%	98.30%	67.9	66.8	98.00%	62.70%	42.6
218	AIS_218	8184.7	95.50%	98.60%	79.8	76.4	94.80%	49.60%	39.5
5	MIA_5	6852.4	95.30%	98.60%	59.3	58.1	97.80%	65.20%	38.7
13	MIA_13	7723.2	94.90%	98.30%	67.1	66	98.10%	66.70%	44.8

14	MIA_14	7966.2	95.00%	98.40%	70	68.3	97.30%	63.60%	44.5
15	MIA_15	6790.8	95.10%	98.40%	59.4	58.3	98.00%	65.20%	38.7
31	MIA_31	7298.8	95.00%	98.40%	63.4	62.2	98.00%	66.30%	42
34	MIA_34	7617.4	95.10%	98.50%	68.2	66.7	97.50%	61.50%	41.9
40	MIA_40	7605.8	95.10%	98.40%	66.4	65.1	97.70%	64.60%	42.9
55	MIA_55	7842	95.00%	98.50%	68.2	66.9	98.00%	65.40%	44.6
69	MIA_69	9291	94.90%	98.40%	81	79.4	97.80%	64.90%	52.6
71	MIA_71	9126.3	94.90%	98.40%	80.4	78	96.60%	62.30%	50.1
76	MIA_76	8460.8	94.80%	98.20%	74	72.3	97.50%	64.30%	47.6
91	MIA_91	7242.9	95.60%	98.70%	62.9	61.8	98.10%	64.70%	40.7
221	MIA_221	9776.9	95.20%	98.60%	87	84.8	97.10%	62.10%	54
23	IAC_23	6011.4	94.90%	98.40%	53	52	97.80%	63.50%	33.6
37	IAC_37	6911.5	94.90%	98.30%	60.8	59.6	97.70%	64.30%	39.1
56	IAC_56	9519.7	95.00%	98.30%	83.7	81.9	97.60%	62.60%	52.4
60	IAC_60	7613.2	95.10%	98.40%	66.2	64.5	97.20%	64.30%	42.6
65	IAC_65	6005.3	95.10%	98.50%	51.6	50.8	98.20%	66.40%	34.2
70	IAC_70	9202.8	95.00%	98.40%	81.7	79.5	97.00%	61.00%	49.8
84	IAC_84	6984.7	94.90%	98.30%	61.6	60.5	98.10%	62.80%	38.7
87	IAC_87	8766.3	95.80%	98.80%	76.1	74.6	97.80%	63.60%	48.4
90	IAC_90	7589.4	94.90%	98.60%	66.9	65.6	97.80%	63.90%	42.8
113	IAC_113	8550.2	95.90%	98.90%	74.6	73.2	97.90%	63.40%	47.3
142	IAC_142	5902.2	94.50%	98.40%	52.4	51.5	97.90%	68.60%	36
150	IAC_150	7638.7	94.70%	98.30%	68.4	67.1	97.90%	64.90%	44.4
207	IAC_207	7737.6	95.20%	98.60%	68.6	67.1	97.50%	63.10%	43.3
208	IAC_208	8931.5	95.30%	98.60%	87.4	83.4	94.30%	47.90%	41.9

Table S13. The correlation between methylation and chromatin accessibility in DARs

Stage	correlation(methylation vs. accessibility)	hyperDARs	hypoDARs
AIS	negative	0	83
	positive	3	29
	all	37	1688
MIA	negative	53	5
	positive	0	0
	all	145	67
IAC	negative	2048	12
	positive	9	7
	all	3996	246
